# Cardiovascular Functions of Ena/VASP Proteins: Past, Present and Beyond

**DOI:** 10.3390/cells12131740

**Published:** 2023-06-28

**Authors:** Peter M. Benz, Timo Frömel, Hebatullah Laban, Joana Zink, Lea Ulrich, Dieter Groneberg, Reinier A. Boon, Philip Poley, Thomas Renne, Cor de Wit, Ingrid Fleming

**Affiliations:** 1 Institute for Vascular Signalling, Centre for Molecular Medicine, Goethe University, 60596 Frankfurt am Main, Germany; 2 German Centre of Cardiovascular Research (DZHK), Partner Site Rhein-Main, 60596 Frankfurt am Main, Germany; 3 Institute of Physiology I, University of Würzburg, 97070 Würzburg, Germany; 4 Cardiopulmonary Institute, 60596 Frankfurt am Main, Germany; 5 Centre of Molecular Medicine, Institute of Cardiovascular Regeneration, Goethe-University, 60596 Frankfurt am Main, Germany; 6 Department of Physiology, Amsterdam Cardiovascular Sciences, VU University Medical Centre, 1081 HZ Amsterdam, The Netherlands; 7 Institut für Physiologie, Universität zu Lübeck, 23562 Lübeck, Germany; 8 German Centre of Cardiovascular Research (DZHK), Partner Site Hamburg/Kiel/Lübeck, 23562 Lübeck, Germany; 9 Institute of Clinical Chemistry and Laboratory Medicine, University Medical Center Hamburg-Eppendorf, 20246 Hamburg, Germany; 10 Center for Thrombosis and Hemostasis (CTH), Johannes Gutenberg University Medical Center, 55131 Mainz, Germany; 11 Irish Centre for Vascular Biology, School of Pharmacy and Biomolecular Sciences, Royal College of Surgeons in Ireland, D02 VN51 Dublin, Ireland

**Keywords:** Ena/VASP proteins, actin dynamics, receptor trafficking, endothelial barrier function, angiogenesis, leukocyte infiltration and polarization, cardiomyocyte contraction, gap junction assembly, conducted vasodilation, smooth muscle cell relaxation

## Abstract

Actin binding proteins are of crucial importance for the spatiotemporal regulation of actin cytoskeletal dynamics, thereby mediating a tremendous range of cellular processes. Since their initial discovery more than 30 years ago, the enabled/vasodilator-stimulated phosphoprotein (Ena/VASP) family has evolved as one of the most fascinating and versatile family of actin regulating proteins. The proteins directly enhance actin filament assembly, but they also organize higher order actin networks and link kinase signaling pathways to actin filament assembly. Thereby, Ena/VASP proteins regulate dynamic cellular processes ranging from membrane protrusions and trafficking, and cell-cell and cell-matrix adhesions, to the generation of mechanical tension and contractile force. Important insights have been gained into the physiological functions of Ena/VASP proteins in platelets, leukocytes, endothelial cells, smooth muscle cells and cardiomyocytes. In this review, we summarize the unique and redundant functions of Ena/VASP proteins in cardiovascular cells and discuss the underlying molecular mechanisms.

## 1. Introduction

**Figure 1:**
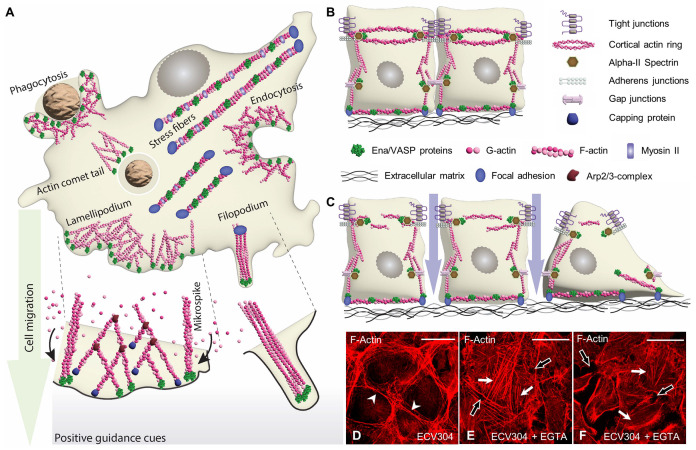
**Actin-polymerization-driven processes in eukaryotic cells.** Directed actin polymerization is the driving force for many cellular processes that shape and move cellular membranes. These processes include cell migration and contraction, endocytosis, phagocytosis and intracellular vesicle trafficking (**A**), as well as the assembly and disassembly of cell-cell junctions and cell-matrix adhesions (**B**–**F**). Regulated by a plethora of actin binding proteins, including the Ena/VASP proteins (green), dynamic actin polymerization generates higher order actin networks and membrane protrusions (magenta). These include the rod-shaped actin stress fibers required for cell contraction; rod-shaped filopodia and microspikes, and branched lamellipodia crucial for cell migration; as well as the branched actin networks in endocytic/phagocytic membrane invaginations and actin comet tails regulating intracellular vesicle transport (**A**). In endothelial cells (**B**–**F**), the formation of the cortical actin ring stabilizes tight and adherens junctions, thereby limiting paracellular permeability and vascular leakage (**B**,**D**). Inflammatory mediators and vasoactive substances induce the transition of the cortical acting ring into contractile stress fibers, which destabilize cell-cell junctions and pull opposing plasma membranes apart to increase paracellular permeability (**C**,**E**,**F**). In confluent ECV304 endothelial cells, actin dynamics form a cortical actin ring that lines the cytoplasmic face of cell-cell contacts (**D**, arrowheads). Following complexation of extracellular calcium by EGTA (**E** 15 min, **F** 30 min), perijunctional actin rings disassemble, focal adhesion associated stress fibers form (**E**,**F** white arrows), and interendothelial junctions open (**E**,**F** black arrows). Scale bars: 20 μm.

Actin dynamics play a vital role in a tremendous range of cellular processes. These processes include the formation of membrane protrusions during cell migration, formation of cell-cell junctions and cell-matrix adhesions, regulation of cell shape and contraction, different forms of exo- and endocytosis and intracellular vesicle trafficking (reviewed in [1,2,3,4,5,6]) (Figure 1). 

In cells, there is a dynamic equilibrium between globular/monomeric (G) actin and filamentous/polymeric (F) actin. Stemming from the appearance of myosin-decorated actin fibers, the fast-growing end is denoted the barbed end (or + end), while the slow-growing end is referred to as the pointed end (or − end) [3]. At least two aspects contribute to the incredible versatility of the actin molecule. First, actin is not a single entity; mammals have six genes that encode closely related actin isoforms: two striated muscle specific isoforms (α-skeletal, α-cardiac), two smooth muscle specific isoforms (α-smooth, γ-smooth), and two ubiquitously expressed cytoplasmic isoforms (β-cytoplasmic, γ-cytoplasmic) [2]. Second, there is an armada of actin binding proteins, that regulate the assembly and disassembly of actin filaments (reviewed in [3,4]). Such proteins bind/sequester monomeric actin and nucleate/polymerize actin filaments, while they also cap and sever actin fibers. Actin binding proteins also branch, cross-link, and bundle actin filaments to form higher order actin networks and membrane protrusions, including stress fibers, the sheet-like lamellipodia or the finger-like filopodia in migrating cells, or the circumferential cortical actin ring/belt underlying cell-cell junctions (Figure 1). The activity of the actin binding proteins is, in turn, tightly regulated by signaling cascades that translate extracellular signals and guidance cues into concerted cellular responses, e.g., directed cell migration and pathfinding.

## 2. Ena/VASP Proteins

One important class of actin binding proteins is the enabled/vasodilator-stimulated phosphoprotein (Ena/VASP) family. Ena/VASP proteins are crucial regulators of the cytoskeleton, linking various kinase signaling pathways to actin assembly [7]. VASP, the founding member of the family, was originally identified and isolated as a target of cAMP-dependent protein kinase (PKA) and cGMP-dependent protein kinase (PKG) in platelets [8,9]. Based on sequence similarity, two additional mammalian family members were identified, EVL (Ena/VASP-like protein) and Mena (mammalian Ena) [10]. While no splice isoforms of the 380 amino acid VASP protein (residues of the human protein) have been reported, alternative splicing generates two EVL isoforms, a short (397 amino acids) and a 21 amino acids longer variant (EVL-I, [11]). Mena has by far the most complex gene structure and in both mice and humans, multiple spice isoforms of the 570 amino acid Mena protein have been identified. This includes three splice variants (Mena^INV^, Mena^11a^ and MenaΔv6) that have been linked to cancer cell invasiveness, and a substantially larger neuronal splice variant, which is also expressed in heart tissue [10,12,13,14,15,16,17].

**Figure 2:**
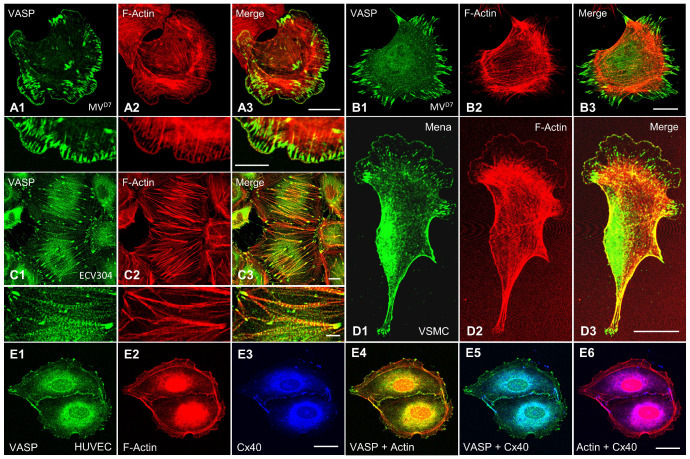
**Subcellular localization of Ena/VASP proteins at sites of high actin turnover.** (**A**,**B**) VASP in transfected Ena/VASP-deficient fibroblasts (MV^D7^) localized predominantly to the leading edge of lamellipodia and tips of microspikes within the lamellipodia (A, magnified views), and at the tips of filopodia (**B**). (**C**) In stably adherent ECV304 endothelial cells, VASP is clearly seen at integrin-based focal adhesions, which anchor actin stress fibers to the extracellular matrix. However, VASP also decorates stress fibers themselves in a punctate pattern (see magnified views). (**D**) Subcellular distribution of Mena in freshly isolated vascular smooth muscle cells (VSMCs) from mouse aorta. (**E**) In human umbilical vein endothelial cells (HUVECs), VASP colocalizes with actin and the gap junction protein connexin 40 (Cx40) at interendothelial junctions. Scale bars: 20 μm; magnified views: 10 μm.

Ena/VASP proteins are concentrated at sites of high actin turnover (compare Figure 1). In motile fibroblasts, endothelial cells and vascular smooth muscle cells, Ena/VASP proteins localize predominantly to the leading edge of branched lamellipodia and the tips of the rod-shaped microspikes (within the lamellipodia) and filopodia (Figure 2A,B,D) [18,19], which are crucial for cell migration and endothelial pathfinding [20]. In platelets, VASP similarly localizes at actin-rich protrusions reminiscent of focal adhesions, filopodia, and lamellipodia upon platelet spreading on fibronectin [21]. In stably adherent endothelial cells and fibroblasts, Ena/VASP proteins are strongly associated with the integrin-based focal adhesions, which anchor actin stress fibers to the extracellular matrix. However, the proteins also decorate the stress fibers themselves in a punctate pattern [18] (Figure 2C). At cell-cell contacts, VASP colocalizes with marker proteins of tight-, adherens- and gap-junctions, and the proteins critically contribute to intercellular adhesion and communication [22,23,24] (Figure 2E). Similarly, Mena and VASP are enriched at intercalated discs, where they contribute to mechanical and electrical coupling of cardiomyocytes [12]. In bone marrow derived macrophages, VASP colocalized with chemokine receptor CCR2 in vesicle-like structures, indicating a function of VASP in CCR2 endocytosis and trafficking [25]. Similarly, Mena was detected in clathrin-coated vesicles during actin-driven epidermal growth factor receptor endocytosis [26]. 

## 3. Domain Organization and Protein–Protein Interactions of Ena/VASP Proteins

**Figure 3:**
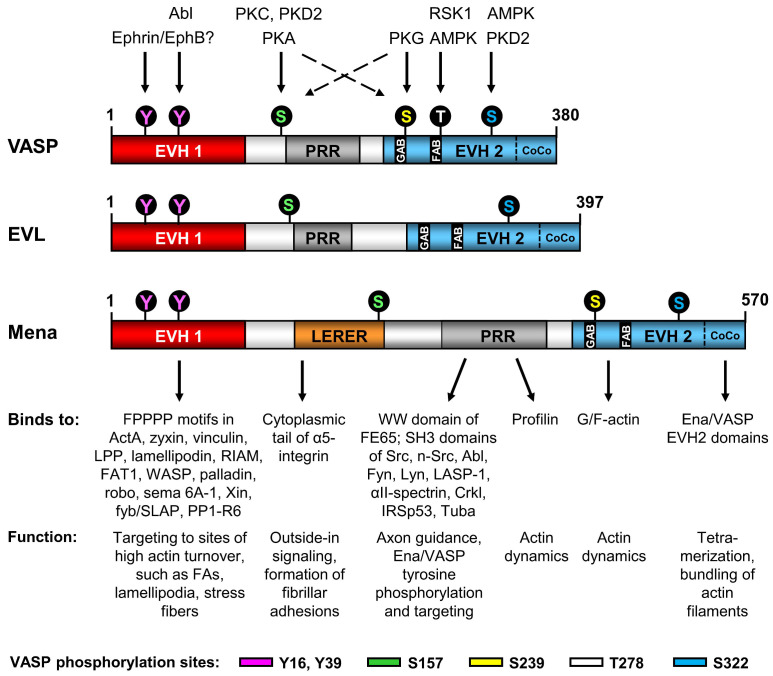
**Domain organization and phosphorylation sites of Ena/VASP proteins along with their binding partners and associated functions.** EVH1: Ena/VASP homology 1, PRR: proline-rich region, LERER: low complexity region harbouring LERER repeats (unique to Mena), EVH2: Ena/VASP homology 2, GAB: G-actin binding site, FAB: F-actin binding site, CoCo: coiled coil motif required for tetramerization. Numbering according to the predominant human protein isoforms. Serine, threonine and tyrosine phosphorylation sites and the respective kinases are also indicated. Except for VASP S239 (which is present in Mena but not in EVL) and T278 (which is unique to VASP), all phosphorylation sites are structurally/functionally conserved in the Ena/VASP protein family (color-coded circles).

Ena/VASP proteins share a tripartite domain organization, consisting an N-terminal Ena/VASP homology 1 (EVH1) domain, a central proline-rich region (PRR), and an EVH2 domain at the C terminus (Figure 3). The EVH1 domain mediates binding of Ena/VASP proteins to proline rich ligands, such as vinculin, zyxin and lamellipodin, thereby targeting them to sites of high actin turnover. The PRR interacts with the actin-binding protein profilin and with Src homology 3 (SH3) domains, whereas the EVH2 domain binds to G- and F-actin and mediates tetramerization of the proteins (Figure 3). Ena/VASP proteins are processive actin polymerases. However, they also bundle actin fibers, antagonize the capping of elongating filaments, and regulate the activity of other actin-binding proteins, such as the Arp2/3, thereby promoting the formation of lamellipodia, microspikes, filopodia, and focal adhesions to positively regulate cell migration and spreading [27,28,29,30,31,32,33]. Ena/VASP proteins also play an important role in actin driven epithelial cell-cell adhesion and endothelial barrier function [22,34,35,36]. During epithelial cell-cell adhesion, two neighboring cells send out actin-based filopodia, which project into the opposing cell’s membrane. Ena/VASP proteins are found at the tips of protruding filopodia and essential for actin dynamics and force generation. After anchoring of the tips, actomyosin contraction creates the reverse force to pull the two cell surfaces together and radial actin fibers reorganize to seal cell borders. If Ena/VASP function is blocked, cell membranes cannot seal [34]. Similarly, Ena/VASP-deficient endothelial cells fail to establish an effective endothelial barrier and demonstrate increased paracellular permeability [22,37,38]. Cardiomyocyte communication via gap junctions is also impaired if the proteins are lacking [12].

**Figure 4:**
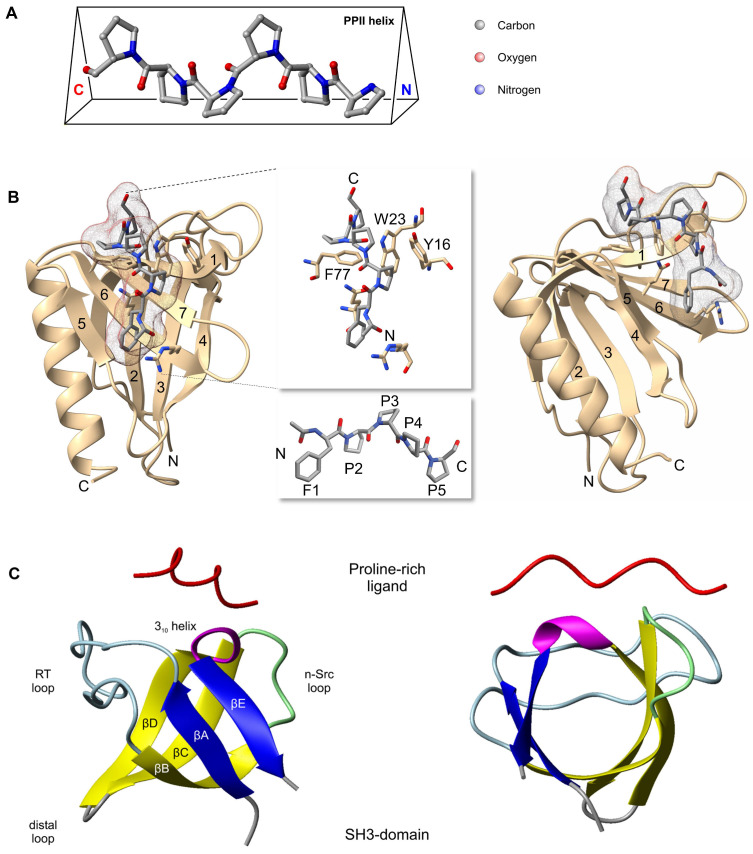
**EVH1 and SH3 domain-mediated protein-protein interactions of Ena/VASP proteins.** (**A**) Ball/stick model of a typical polyproline type II (PPII) helix as determined by X-ray diffraction (modified from pdb ID: 1FYN). PPII are left-handed helices that can be represented as triangular prism. Three consecutive prolines account for one turn of the PPII helix and occupy a different edge of the prism. (**B**) Ribbon diagram of the Mena EVH1 domain in complex with a FPPPP core peptide ligand. The overall fold of EVH1 domains is a compact, parallel β-sandwich capped along one side by a long α-helix. The highly conserved triad of surface-exposed aromatic sidechains, Y16, W23, and F77 (F79 in VASP), come together in the 3-dimensional structure of the domain to form an aromatic cluster, which provides a hydrophobic docking site for the proline-rich peptide ligands (modified from pdb ID: 1evh). (**C**) Ribbon model of chicken α-spectrin SH3 domain as determined by X-ray diffraction (pdb ID: 1SHG) in a hypothetical complex with a proline-rich ligand (PPPVPPRV, pdb ID: 1CKB). The SH3 domain is a compact β-barrel made of five antiparallel β-strands (βA-βE) that are connected (from N- to C-terminus) by the RT, n-Src, and distal loop, and by a 3_10_ helix, respectively. β-Strands βA and βE, and β-strands βB-βD form two tightly packed anti-parallel β sheets that are shown in blue or yellow, respectively. The β-barrel is shown in two different orientations related by a 90° rotation.

Short proline-rich motifs are directly involved in protein-protein interactions, regulating signal transduction, vesicular trafficking and cytoskeletal dynamics. Proline is unique among the 20 naturally occurring amino acids, having a cyclic side chain connecting to the backbone nitrogen atom. This arrangement restricts the conformation of the proline itself as well as adjacent residues. As a consequence, proline-rich sequences adopt a left-handed polyproline II (PP II) helix, with three residues per turn. With its triangular cross-section, the aliphatic side chains of the PP II helix form a hydrophobic surface, while the backbone carbonyls present ideal hydrogen bond acceptors [39,40] (Figure 4A). Because proline-rich regions are easily accessible, their on-and off-rates for binding can be very fast, allowing them to facilitate the rapid assembly and disassembly of protein complexes [39]. Proline-recognition domains (PRDs) include SH3 domains, WW domains, EVH1 domains and the profilin proteins [40,41,42]. The protein-protein interactions of Ena/VASP proteins are largely dominated by proline-rich sequences and the respective PRDs. Mena, VASP and EVL contain an N-terminal EVH1 domain and, via their central proline-rich region, have been shown to directly interact with SH3 domains, WW domains and profilin.

### 3.1. EVH1 Domain

EVH1 domains are ~115 residue noncatalytic protein-protein interaction modules, found in a large number of multi-domain proteins that are often involved in modulating the actin cytoskeleton or in signal transduction. Four protein families contain EVH1 domains: the Ena/VASP proteins, the Wiscott-Aldrich syndrome (WASP, N-WASP) proteins, the Homer/Vessel synaptic scaffolding proteins, and the Sprouty-related proteins with an EVH1 domain (SPRED) [40,41,43]. The overall fold of EVH1 domains, which are exclusively found at the N-terminus of host proteins, is very similar to pleckstrin homology (PH) domains, forming a compact, parallel β-sandwich capped along one side by a long α-helix [44,45]. A specific feature of EVH1 domains is the highly conserved triad of surface-exposed aromatic sidechains, Y16, W23, and F79 (numbering of human VASP). These three residues come together in the 3-dimensional structure of the domain to form an aromatic cluster, which provides a hydrophobic docking site for proline-rich peptide ligands [43,44] (Figure 4B). The Ena/VASP EVH1 domain binds to so-called FPPPP motifs, proline rich peptides with the core consensus sequence (**F**/W/L/Y)PxφP (where x denotes any amino acid and φ a hydrophobic residue [46,47]). While a tryptophane residue at the first position shows an increased affinity to EVH1 domains in solid phase binding assays, phenylalanine is the dominating residue in naturally occurring EVH1 ligands [40,47].

The first EVH1/FPPPP ligand identified was the actin assembly-inducing protein (ActA) of the bacterial pathogen Listeria monocytogenes. In cells, the ActA protein is essential to recruit the host cell’s actin nucleation and polymerization machinery, including Ena/VASP proteins, which assembles a so-called actin comet tail on the bacterial surface and in turn drives bacterial propulsion [32,47,48,49]. Subsequently, a number of functional ActA-like repeats were also identified in host cell proteins that are associated with sites of high actin turnover. This includes the focal adhesion proteins zyxin, vinculin and LPP [47,50,51,52,53], the lamellipodia/leading edge proteins lamellipodin [54], Rap1-GTP-interacting adaptor molecule (RIAM) [55], protocadherin FAT1 [56], and WASP [57], as well as the stress fiber associated protein palladin [58]. Further ActA-like motifs are found in the two transmembrane guidance cue molecules, namely roundabout [59] and semaphoring 6A-1 [60], the filamin-c binding protein Xin [61], the fyn-binding and SLP-76 associated protein (fyb/SLAP) [62], which is important during T-cell activation, as well as the regulatory subunit 6 of protein phosphatase 1 (PP1-R6) [63]. The Ena/VASP EVH1 domain has also been shown to directly interact with Abi1, a component of the WAVE regulatory complex [64]. Of all the protein interactions with Ena/VASP EVH1 domains, the interaction with Tes, a focal adhesion protein, stands out as it breaks the rule for EVH1 domains. Tes lacks a proline rich FPPPP motif and the interaction with the EVH1 domain of Mena (but not VASP or EVL) is mediated via its C-terminal LIM3 domain [65].

Interactions of EVH1 domains with FPPPP motifs are important for the subcellular targeting of Ena/VASP proteins (reviewed in [19]) but the core motif alone is not sufficient for efficient binding and core-flanking epitopes are required to achieve the necessary specificity. For example, the binding affinity of the ActA peptide D**FPPPP**TDEEL to the Mena EVH1 domain can be reduced 100-fold by truncation of core-flanking residues to generate a **FPPPP**T peptide [45]. Notably, several of the established Ena/VASP EVH1-domain interacting proteins, including ActA, zyxin and lamellipodin, contain several closely-spaced FPPPP motifs. Given that Ena/VASP proteins form tetramers via their C-terminal EVH2 domain (see below), this potentially allows for avidity effects in complex formation, e.g., two or more Ena/VASP proteins of the same tetramer synergistically binding to a target protein with several FPPPP motifs. Indeed, isolated EVH1 domains fail to robustly translocate to lamellipodia and focal adhesions in transfected cells [66]. However, EVH1 domains can mediate targeting to these subcellular sites when combined with the tetramerization motif from the EVH2 domain [19].

Harnessing the ActA-mediated recruitment of Ena/VASP proteins in cells, the EVH1 domain mediated interaction of Ena/VASP proteins has also been used in numerous functional studies to neutralize Ena/VASP activity. Exogenous expression of the high affinity FPPPP motifs of ActA coupled to EGFP and a sequence that targets the fusion protein to the outer mitochondrial membrane (EGFP-FP4-mito) was used to sequester Ena/VASP proteins away from their subcellular sites of function. Replacing the critical phenylalanine by an alanine (EGFP-AP4-mito), which abrogates EVH1 binding, served as control in these experiments [67,68]. Recently, nanomolar small-molecule inhibitors of Ena/VASP EVH1 domains have been developed to target invasion and extravasation of breast cancer cells [69].

### 3.2. LERER Repeats

Mena modulates the bi-directional signaling between the extracellular matrix and the actin cytoskeleton [70]. Unlike VASP and EVL, Mena contains a 91-residue low complexity region with 13 repeats of the five amino acid motif LERER, C-terminal to the EVH1 domain (Figure 3). The LERER repeats bind directly to the cytoplasmic tail of *α*5 integrin. In fibroblasts, the Mena–*α*5 complex is required for “outside-in” *α*5β1 signaling, including the phosphorylation of FAK and paxillin as well as formation of fibrillar adhesions. The motif is also required for fibrillogenesis and cell spreading, and regulates cell migration speed [70].

### 3.3. Proline-Rich Region

**Figure 5:**
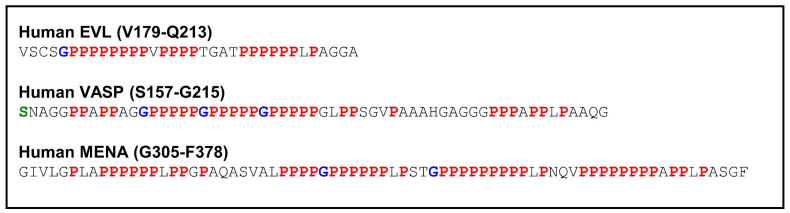
**Comparison of the proline-rich regions from human EVL, VASP, and Mena.** Prolines are shown in red, glycines preceding a proline-stretch in blue. The preferred PKA phosphorylation site in VASP, S157 (green), is located in close proximity to the GP_5_ motifs. The PRR of Mena is the largest, spanning 64 amino acids, followed by VASP with 50, and EVL with only 25. EVL contains a single GP_8_ motif, VASP a triple GP_5_ motif, and Mena a GP_6_ and GP_9_ motif.

While the EVH1 and EVH2 domains are well conserved among the Ena/VASP proteins, the proline-rich region (PRR) is the most divergent and may differ in binding modes and mechanisms of regulation [71]. The PRR of Mena is the largest, spanning 64 amino acids, followed by that of VASP with 50, and EVL with only 25 residues. The PRRs are characterized by motifs of a glycine residue followed by multiple prolines. EVL contains a single GP_8_ motif, VASP a triple GP_5_ motif, and Mena a GP_6_ and GP_9_ motif (Figure 5). Furthermore, the spacing between the preferred PKA phosphorylation site and the GP_x_ motifs of the different family members is quite different. While the PKA phosphorylation site in VASP is close to the triple GP_5_ motif (S157 vs. G169, human VASP), the distance is substantially greater in EVL (S160 vs. G183, human EVL) and in Mena (S265 vs. G335, human 570 residue Mena variant).

The G-actin binding protein, profilin, was the first identified ligand of the PRR in the Ena/VASP proteins [72]. Initially, profilin was reported to bind to the triple GP_5_ motif of VASP with relatively low affinity [73]. However, later studies suggested that the high-affinity profilin/VASP binding peptide ^198^GAGGGPPPAPPLPAAQ^213^ is located more C-terminal within the PRR [74]. Whether or not both binding sites exist in parallel is currently unclear. Consistent with the latter study, however, profilin did not interfere with the binding of the αII-spectrin SH3 domain to the triple GP_5_ motif of VASP [22]. Interestingly, profilin-actin complexes bind to the VASP PRR with substantially higher affinity than profilin alone, at least in vitro [74]. Given that profilin-actin complexes constitute the major pool of polymerization-competent actin in cells, these findings suggest an important contribution of Ena/VASP–profilin interactions for actin assembly. However, there are currently conflicting reports, either supporting or contradicting the hypothesis that Ena/VASP proteins utilize profilin to accelerate actin assembly in vivo [19,29,75,76,77,78].

The PRR of ENA/VASP proteins can also associate with WW domains in other proteins. One example is the interaction of the Mena PRR with the WW domain of amyloid-β A4 precursor protein-binding family B member 1 (APBB1, also referred to as FE65), which plays a central role in axonal growth cone dynamics, axon guidance, and neuronal positioning in the developing brain [79,80].

An interesting aspect of the Ena/VASP PRRs is their association with SH3 domains. Mostly found in signal transduction and cytoskeletal proteins, these 60-residue protein modules have a characteristic fold: a compact β-barrel, formed by five β-strands that are arranged as two tightly packed anti-parallel β sheets [42,81,82] (Figure 4C). Ena/VASP proteins have been shown to bind to a variety of SH3 domains, including the non-receptor tyrosine kinases Src, neuronal-Src, Abl, Fyn and Lyn [10,11,83,84]. However, they also interact with the SH3 domains of the actin binding protein LASP-1 [85], the membrane skeleton protein αII-spectrin [12,22,86], and the scaffold protein IRSp53 that couples membranes with the cytoskeleton in actin-rich protrusions such as filopodia and lamellipodia [87,88]. In platelets, the N-terminal SH3 domain of the cytoskeletal and transcriptional regulator CrkL was found to bind VASP [21], and the scaffold protein Tuba, which links dynamin to the actin cytoskeleton, was shown to directly bind to Ena/VASP proteins via its C-terminal SH3 domain [89]. Consistent with the low sequence conservation of the PRRs between Ena/VASP proteins, SH3 binding partners differ between the individual family members. For example, EVL binds to the SH3 domains of Lyn, neuronal-Src and Abl, whereas Mena only binds to Abl and Src SH3 domains, but not to Lyn or neuronal-Src [10,11]. Notably, the alternative splice ‘+ Exon’ of murine and human Mena, which introduces an additional 242 or 228 amino acids, respectively, contains long proline-rich stretches with further putative SH3 domain binding sites, including a PPTPPLR motif that conforms with the consensus sequence of classical SH3 ligands (see below and [17]). While interaction with SH3 domains of the scaffold/cytoskeletal proteins mediates subcellular targeting of Ena/VASP proteins to orchestrate actin dynamics, the interactions with Src, neuronal-Src, Abl, Fyn and Lyn seem related to the tyrosine phosphorylation of the Ena/VASP proteins. Indeed, the Abl-dependent tyrosine phosphorylation of Mena and VASP has been reported and bridging of the proteins by Abl interactor 1 (Abi-1) appears to foster the effect [90,91]. On the other hand, the interaction of Ena/VASP proteins with Abl has been suggested to regulate the activity of the tyrosine kinase, as the intramolecular binding of the Abl SH3 domain is important to hold Abl in a catalytically inactive conformation [84].

None of the PRRs within the Ena/VASP proteins contain a positively charged residue that is seen in classical SH3 ligands and the interactions appear to be mediated by atypical SH3 domains. The latter, including Abl and the αII-spectrin SH3 domain, prefer ligands, in which a hydrophobic amino acid replaces the positive charged residue [92]. SH3 domain-mediated interactions are frequently implicated in processes that require the rapid subcellular recruitment or interchange of proteins during initiation of signaling cascades and cytoskeletal rearrangements [39]. In cells, several mechanisms exist to determine protein binding selectivity. These are temporal and cell-type specific gene expression, combination of multiple separate interactions between two binding partners, and the cooperative assembly of multiprotein complexes. Most common, however, is the compartmentalization of binding partners and the regulation of their interaction by posttranslational modifications such as phosphorylation [39,92]. Indeed, several SH3-domain dependent interactions with Ena/VASP proteins have been shown to be negatively regulated by PKA activity. For example, PKA-mediated phosphorylation of EVL abrogates its interaction with Abl and neuronal-Src [11]. Similarly phosphorylation of VASP inhibits its binding to the SH3 domains of Abl, CrkL and αII-spectrin [12,21,22,84]. In contrast, complex formation of Ena/VASP proteins with the SH3 domain of Lyn, the WW domain of FE65 and with profilin is independent of the PKA-mediated phosphorylation status of Ena/VASP proteins [11,93].

### 3.4. EVH2 Domain

The EVH2 domain of Ena/VASP proteins contains the G-actin binding site (GAB), the F-actin binding site (FAB) and a short right-handed coiled-coil (CoCo) motif that is required for the tetramerization of the proteins [94,95,96] (Figure 3). The GAB ^223^APGLAAAIAGAKLRKV**S**^239^ (underlined sequence, numbering relative to human VASP; the preferred PKG phosphorylation site S239 is bold) resembles the 16-19 amino acid WH2 motif, which binds to the hydrophobic cleft of actin and is found in many additional actin binding proteins [97]. Within this sequence, the basic KLRK motif is crucial for G-actin binding and very similar to the G-actin binding motif found in thymosin β4 (KLKK) [96,98]. First evidence for a direct interaction of Ena/VASP proteins with actin filaments was obtained by co-sedimentation assays of VASP and F-actin [99]. Later, the FAB was mapped to an amino acid stretch i.e., 259-276 ^259^GGGGLMEEMNAMLARRRKA**T**^278^ (underlined sequence, numbering relative to human VASP; the preferred AMPK phosphorylation site T278 is bold) within the EVH2 domain and the tetramerization motif of the Ena/VASP proteins was located at the extreme C-terminus of the protein (343-380). Together, FAB and the tetramerization domain are essential to bundle actin fibers [94]. The role of VASP and the EVH2 domain was confirmed in a classic in vitro actin polymerization assay. Addition of wild-type VASP (or the EVH2 domain alone) increased in vitro actin polymerization markedly [18,96,100].

In cells, early studies reported conflicting results and the role of Ena/VASP proteins in actin assembly has been controversially debated for almost a decade [101]. Only with advanced imaging techniques, studies using Dictyostelium VASP, which is characterized by a high affinity GAB motif, and the use of quantitative mathematical modeling, it has been possible to better understand the molecular mechanism of Ena/VASP-mediated actin filament elongation [28,29,75]. One of the Ena/VASP EVH2 domains is associated with the barbed end of the growing actin filament, while the remaining three EVH2 domains from the Ena/VASP tetramer are thought to be fully saturated with G-actin and enhance filament elongation by transferring G-actin onto the barbed end. After G-actin release, the ‘new’ EVH2 domain remains associated with the end of the growing filament while the previously filament associated, non-terminal EVH2 domain, is released to recruit a new monomeric actin subunit and to continue actin polymerization [19,28]. Furthermore, the actin polymerization activity of the Ena/VASP proteins at the barbed end also delays association of capping proteins, thereby further promoting filament elongation [29,31].

## 4. Regulation of Ena/VASP Proteins by Phosphorylation

Ena/VASP proteins are well-established targets of serine, threonine, and tyrosine protein kinase (PK) pathways [18,102] (Figure 3). Functionally, phosphorylation events have been shown to control the subcellular targeting of Ena/VASP proteins and their ability to modulate actin dynamics, at least in part by modulating their protein-protein interactions [18,22,103]. Notably, the phosphorylation status of VASP is frequently used to assess PKA/PKG-dependent effects in cardiovascular cells as well as platelet reactivity [104,105,106,107,108]. To date, ~30 potential serine/threonine/tyrosine phosphorylation sites have been identified by high throughput mass spectrometry in each of the human proteins (www.phosphosite.org, accessed on 25 April 2023). However, most sites have yet to be confirmed experimentally. The most prominent tyrosine phosphorylation sites in human VASP are Y16 and Y39 within the EVH1 domain (both conserved in EVL and Mena). Serine phosphorylation sites include S157 in the PRR (corresponding to EVL S160 and Mena S265), S239 in the EVH2 domain in immediate proximity to the GAB motif (Mena S405, not conserved in EVL), and S322 in the EVH2 domain between the FAB and the tetramerization motif (EVL S362 and Mena S512). The most prominent threonine phosphorylation site in VASP is T278 in the EVH2 domain in immediate proximity to the FAB motif (not conserved in EVL or Mena) (Figure 3). Among the Ena/VASP proteins, phosphorylation of VASP is by far best studied, at least partially due to the availability of excellent (phosphospecific) antibodies, which are frequently used to monitor the activation of kinase signaling pathways [107,108].

### 4.1. Ena/VASP Serine/Threonine Phosphorylation

S157 is the preferred phosphorylation site of PKA, which can also phosphorylate S239, while PKG preferentially phosphorylates S239 and then S157 [109,110]. PKD1 has also been shown to phosphorylate S157 (and S322) in vitro and in living cells [111], but a later study by the same group showed that the enzyme PKD2 is more likely to phosphorylate S157 and S322 [112]. Given that PKD is activated downstream of PKC and ROCK1, PKD-mediated VASP phosphorylation at S157 may also explain previous findings regarding the PKC- and the thrombin/ROCK1-dependent phosphorylation events at this site [113,114]. T278 is phosphorylated by the AMP-activated protein kinase (AMPK) [103], which also contributes to S322 phosphorylation [115], and by ribosomal S6 kinase 1 (RSK1, [116]). Phosphorylation of VASP on S157, but none of the other phosphorylation sites, leads to a shift in its apparent molecular mass from 46 to 50 kDa in SDS-PAGE [9,103,109]. Although the molecular cause for the shift is not well understood, a similar phenomenon is evident in EVL and in Mena, albeit to a lesser extent [10,11]. While VASP S157 phosphorylation (and the equivalent sites in EVL and Mena) has little impact on actin filament assembly or the G- to F-actin ratio [18], it negatively regulates distinct SH3-domain mediated interactions of VASP or EVL with Abl, neuronal-Src, CrkL and αII-Spectrin [11,12,21,22,84]. Interestingly, S157 phosphorylation does not regulate Ena/VASP interactions with the SH3 domain of Lyn, the WW domain of FE65, or profilin. It also does not affect the EVH1-mediated interaction of Ena/VASP proteins or the EVH2-mediated tetramerization of the proteins [11,93]. Overall, it seems that S157 phosphorylation may represent a molecular switch to facilitate the crosstalk of PKA, PKG and PKD to other kinase signaling pathways (e.g., by interaction with Abl/Src), or to regulate the subcellular distribution of Ena/VASP proteins (e.g., by interaction with αII-spectrin or CrkL). Indeed, VASP S157 phosphorylation drives the subcellular targeting of the protein to the periphery, including the membrane/leading edge or focal adhesions [18,22,111]. So far, no phosphorylation-dependent SH3 domain interactions with Mena have been reported. While the VASP S157 phosphorylation site is functionally conserved in Mena, it is located 70 amino acids N-terminal to the PRR, which harbors the SH3 binding peptides, suggesting that regulation of SH3 domain interactions with Mena may be different from VASP and EVL, where the phosphorylation site is in closer proximity to the PRRs.

An interesting aspect of PKD-mediated VASP phosphorylation is the simultaneous phosphorylation of S157 and S322 downstream of RhoA activation. Phosphorylation of VASP at S157 and S322 by PKD elicits the translocation of VASP from focal adhesions to the leading edge, which increases the number and extension of filopodia [111]. S157 and S322 are both conserved in EVL and Mena, but experimental proof for the PKD-mediated phosphorylation of the proteins is lacking. Nevertheless, the 21 amino acids insert in EVL-I has been reported to be phosphorylated by PKD, which supported filopodia formation in migrating cells [117]. Interestingly, vascular endothelial growth factor (VEGF)-induces PKD phosphorylation and activation and increasing evidence suggests a critical role of PKD in VEGF-induced endothelial cell migration and angiogenesis [118,119]. Thus, it is tempting to speculate that EVL-I executes a specialized role in angiogenesis in response to PKD activation. Indeed, it was recently shown that EVL-I is only expressed in the postnatal, but not in the adult retina and that EVL deficiency significantly impaired sprouting angiogenesis in the postnatal retina [120].

Functionally, phosphorylation and phosphomimetic substitution of S239 and T278 has been reported to synergistically impair VASP-driven actin assembly in vitro and in living cells [18,103]. Supporting the inhibitory role of S239- and T278-phosphorylation in VASP-driven actin assembly, Barzik and colleagues demonstrated that VASP phosphorylation at S239 and T278, but not at S157, decreased its anti-capping and F-actin bundling activities in vitro [31]. Given that actin filaments are negatively charged and S239 and T278 are located in immediate proximity to the GAB and FAB, it is likely that adding a negative charge to these residues by either phosphorylation or phosphomimetic substitution, favors the repulsion of Ena/VASP proteins from actin filaments, and thus reduces their actin polymerization capabilities.

### 4.2. Ena/VASP Tyrosine Phosphorylation and Its Implication in Ephrin/EphB Signaling

Multiple high throughput proteomic studies have identified phosphorylation of Ena/VASP proteins on Y16 and/or Y39 within the EVH1 domain [121,122,123]. So far, however, only VASP Y39-phosphorylation has been experimentally confirmed and phosphorylation by c-Abl or the Bcr-Abl oncogene requires a ternary complex with Abi-1 or Abi-2 [91]. Indeed, the human VASP sequence ^38^I**Y**HNP^42^ fits the general consensus sequence of Abl (I/V/L-**Y**-Xn-P/F, where n is 2 or 3) [124]. To address the functional relevance of Y39-phosphorylation, Y39 was either substituted by phenylalanine (Y39F) or aspartate (Y39D), to generate a non-phosphorylatable and phosphomimetic mutant, respectively. While the intracellular localization of the Y39F mutant was similar to the wild-type protein in fibroblasts, the Y39D mutant was diffusively distributed in the cytoplasm and failed to efficiently localize to focal adhesions. Also, Y39D binding to a GST-fusion protein harboring the FPPPP repeats of zyxin was reduced [91]. While it is possible that phosphorylation events within the EVH1 domain may regulate Ena/VASP binding to established ligands, care must be taken when interpreting the results of the Y39D mutant. In contrast to Y16, which is a residue of the aromatic triad forming the hydrophobic docking site for the proline-rich EVH1 ligands, Y39 is located at the C-terminal end of the second β-sheet of the EVH1 domain, opposite of its hydrophobic docking surface [45] (Figure 4B). Therefore, it seems possible, that substitution of the aromatic Y39 residue by aspartic acid may alter the folding/3D-structure of the EVH1 domain and thus impair zyxin binding, rather than protein function.

**Figure 6:**
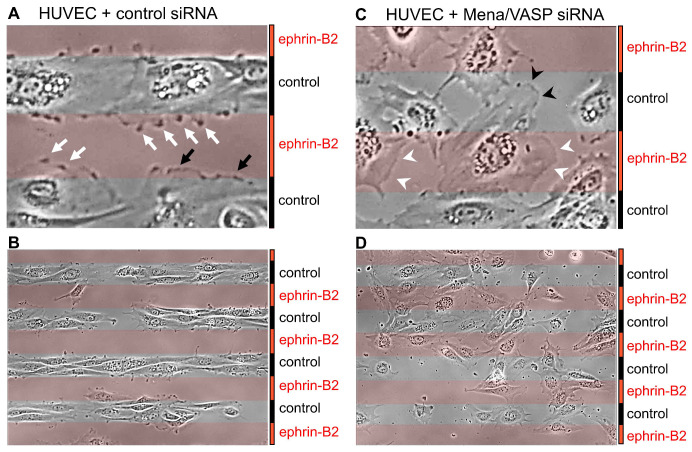
**Role of Ena/VASP proteins in endothelial cell repulsion from ephrin ligands.** HUVECs, treated with control (**A**,**B**) or Mena/VASP (**C**,**D**) siRNA, were seeded on alternating 50 μm stripes with clustered ephrin-B2 (labeled with Cy3 fluorescent dye) or control and imaged by time-lapse microcopy. Representative phase contrast images of cells approximately two hours (**A**,**C**, magnified view) or five hours (**B**,**D**) after seeding are shown; ephrin-B2 stripes are indicated by red overlays. Please note the cytoskeletal collapse in control siRNA-transfected HUVECs on ephrin-B2 stripes, resulting in dot-like structures and membrane ruffles (**A**, white and black arrows, respectively), whereas no cytoskeletal collapse was observed in Mena/VASP siRNA transfected cells on ephrin-B2 stripes (**C**, compare lamellipodia indicated by black and white arrowheads, respectively). Figure modified from [127].

Ena/VASP tyrosine phosphorylation seems also to be implicated in ephrin/EphB signaling in endothelial cells. Endothelial tip cells form filopodia, which sense the environment for guidance cues and thereby determine the direction of growth [20]. A mechanism that regulates vascular development and postnatal angiogenesis is the interaction of the EphB receptor tyrosine kinases with their transmembrane ephrin-B ligands. Ephrin-Eph signaling is probably best known for the discrimination of arterial and venous territories by repulsion of venous endothelial cells away from those with an arterial fate. Ultimately, cell repulsion is mediated by initiating the collapse of the actin cytoskeleton in membrane protrusions [125]. Although Ena/VASP proteins have not yet been implicated in endothelial cell guidance, they are perfectly positioned to translate guidance cues into actin assembly at the leading edge and into directed cell migration (compare Figure 1 and Figure 2). Furthermore, Ena/VASP proteins colocalize with activated EphB receptors and have been implicated in the repulsion of neural crest cells from ephrin ligands [126]. Notably, tyrosine phosphorylated (Y16 and Y39) VASP peptides were detected by mass spectrometry in HEK cells with activated ephrin/EphB signaling [122]. The role of the Ena/VASP proteins in endothelial cell repulsion was tested in vitro using alternating stripes of control or ephrin-B2 coated surfaces. Human endothelial cells dynamically extended sheet-like lamellipodia over ephrin-B2 coated surfaces. While lamellipodia of control siRNA transfected cells rapidly collapsed, resulting in a pronounced cell avoidance of the ephrin-B2 surfaces, the knockdown of Ena/VASP proteins impaired the cytoskeletal collapse of membrane protrusions and the cells no longer avoided the repulsive coating [127] (Figure 6). Mechanistically, ephrin-B2 stimulation elicited the EphB-mediated tyrosine phosphorylation of VASP, which abrogated its interaction with the focal adhesion protein zyxin. Nck2 was identified as a novel VASP binding protein, which only interacted with the tyrosine phosphorylated VASP protein, potentially via the SH2 domain of Nck2. Nck2 links Eph-receptors to the actin cytoskeleton. Therefore, it was hypothesized that Nck2-Ena/VASP complex formation is required for actin reorganization and/or Eph receptor internalization downstream of ephrin-Eph interaction in endothelial cells, with implications for endothelial cell navigation and pathfinding [127]. Interestingly, Nck and VASP have been identified in a macromolecular complex that links the actin cytoskeleton to Fcγ receptor signaling during phagocytosis [128], suggesting that Nck-VASP binding may not be limited to signaling events downstream of Eph-receptors.

## 5. Ena/VASP-Deficient Mouse Models

To elucidate the function of Ena/VASP proteins in vivo, the Mena, VASP, and EVL genes have been disrupted in mice. Mice lacking all three mammalian Ena/VASP proteins were generated, but triple null progeny failed to reach term and died in utero between E16.5 and P0 [129]. Interestingly, a single allele of Mena was sufficient to rescue the embryonic lethal phenotype and to produced viable and fertile mice, albeit at low frequency. In contrast, neither two alleles of EVL nor VASP were sufficient for survival if the other two Ena/VASP genes were disrupted [129], suggesting an indispensable function of Mena during neural development.

Knocking out individual Ena/VASP genes resulted in surprisingly mild phenotypes and Mena^−/−^, VASP^−/−^, and EVL^−/−^ mice were reported to be viable and fertile and macroscopically indistinguishable from wild-type mice [130,131,132]. More specifically, VASP-deficient mice displayed increased numbers of megakaryocytes but platelets were dysfunctional with an impaired cyclic-nucleotide-mediated inhibition of aggregation [130,131,133]. EVL^−/−^ mice showed reduced endothelial tip cell density and filopodia formation at the angiogenic front, which resulted in a compromised radial sprouting of the vascular plexus in the retina during postnatal development [120]. Mena^−/−^ mice were reported to display defects in central nervous system architecture, including misrouted axon projections from interhemispheric neurons. Otherwise, Mena null animals were reported to be fully viable and obtained in the expected Mendelian ratios [132]. Given the essential roles of Ena/VASP proteins, the rather mild phenotypes found in the mutant mice were surprising and argued for a functional compensation of the proteins. Indeed, Ena/VASP double- and triple-deficient mice die early in development and display severe neural tube defects and facial malformations [38,134].

In an alternative approach to target Mena gene expression in vivo, a β-galactosidase/neomycin-based gene trap (GT) vector was inserted into intron 2 of the Mena gene, thereby replacing the Mena protein by a β-galactosidase fusion protein that is expressed under the control of the endogenous Mena promoter. Surprisingly, the gene trap was leaky in neuronal tissue and homozygous Mena^GT/GT^ mice displayed basal Mena protein expression in neuronal tissue, but minimal Mena protein expression in most other tissues. The remaining Mena expression in the brain appeared sufficient to generate viable and fertile VASP^−/−^Mena^GT/GT^ mice that essentially lacked Mena and VASP expression in cardiovascular cells, including blood vessels, lung, and heart [12].

In a recent attempt to generate global Mena knockout mice with the potential to generate tissue-specific Mena^−/−^ animals, ES-cells from the European Conditional Mouse Mutagenesis Program (Enah^Gt(EUCE322f03)Hmgu^, parent cell line: E14TG2a) were used to generate chimeras by injections of C57BL/6 host blastocysts and subsequently heterozygous Mena^+/F03^ animals. Surprisingly and in contrast to a previous report [132], bi-allelic ablation of Mena in homozygous mutant animals (Mena^F03/F03^) resulted in embryolethality and mice died in utero [135]. E11 Mena^F03/F03^ embryos were small and runted and displayed craniofacial defects, reminiscent of Mena/VASP-double deficient animals [134]. Currently, it remains unclear why Mena deletion induced such different phenotypes in two different founder lines. Potential explanations include incomplete Mena gene inactivation, e.g., due to splicing events, or the varying genetic background of the mutant mice.

## 6. Role of VASP in Platelets

Platelets are the second most common blood cells in the human body. Along with the coagulation factors, the main function of platelets is to stop bleeding by aggregating and sealing blood vessel injuries. Nitric oxide (NO) and prostacyclin play a crucial role in preventing platelet adhesion and aggregation. They activate soluble guanylate cyclase and adenylate cyclase, respectively, initiating a subsequent increase in cGMP and cAMP, to activate PKG and PKA. Targets of the latter kinases inhibit events normally associated with platelet activation, such as the elevation of intracellular calcium, integrin activation, cytoskeletal reorganization, and platelet granule secretion [108]. Of the Ena/VASP proteins, only VASP seems to be expressed in platelets [136]. Activation of VASP-deficient platelets with thrombin elicited a significantly increased P-selectin expression and fibrinogen binding compared to wild-type controls, indicating that VASP is a negative regulator of integrin a_IIb_β_3_ activation [131]. Furthermore, the cAMP- and cGMP-mediated inhibition of platelet aggregation was significantly reduced in the absence of VASP, but cytosolic calcium concentrations and granule secretion were unaffected by VASP deletion [130]. Platelet–vessel wall interactions were, as a consequence, significantly enhanced in VASP^−/−^ mice [133].

How VASP negatively controls platelet integrins is not entirely clear. Since activation of α_IIb_β_3_ integrin is dependent on the small GTPase Rap1b [137,138,139], it was hypothesized that VASP may be a negative regulator of α_IIb_β_3_ integrin through its ability to inhibit Rap1b [21]. However, the activation of Rap1b in response to thrombin, ADP, or thromboxane A2 receptor agonists was reduced rather than enhanced in platelets from VASP-null mice. Impaired activation of Rap1b in VASP-null platelets was due neither to changed expression levels of guanine nucleotide exchange factors (GEFs) or GTPase-activating proteins (GAPs), which activate or inactivate Rab1b, respectively, nor to defects in the redistribution of the proteins between cytosolic and membrane fractions. Rather the phenomenon could be attributed to the association between VASP and the Crk-like protein (CrkL), an adapter protein, which activates the Rap1b guanine nucleotide exchange factor C3G [140,141]. CrkL co-immunoprecipitated VASP from platelet lysates and CrkL and VASP dynamically co-localized at actin-rich protrusions reminiscent of focal adhesions, filopodia, and lamellipodia upon platelet spreading on fibronectin. Recombinant VASP bound directly to the N-terminal SH3 domain of CrkL and PKA-mediated VASP phosphorylation on S157 abrogated the binding to CrkL [21]. Overall, it seems that the formation of a ternary C3G/CrkL/VASP complex regulates Rap1b-dependent platelet activation and this may explain why agonist-induced activation of Rap1b in VASP-null platelets is impaired. PKA-dependent phosphorylation of VASP on S157 abrogated CrkL binding, which may provide, at least in part, a rationale for the PKA-dependent inhibition of Rap1b activation and platelet aggregation [21].

In addition to its role in platelet adhesion and aggregation, VASP is also implicated in the formation of platelet-neutrophil complexes (PNCs), which aggravate inflammatory tissue injury [142,143]. In vitro, phosphorylation of VASP reduced the formation and transendothelial movement of PNCs. During myocardial ischemia reperfusion (IR) injury, hematopoietic VASP expression was found to be crucial for the intravascular formation of PNCs, the presence of PNCs within ischemic myocardial tissue and the extent of myocardial IR injury. Conversely, phosphorylation of VASP on the preferred PKA and PKG phosphorylation sites reduced intravascular PNC formation and presence of PNCs within ischemic myocardial tissue [143]. Later studies revealed that VASP phosphorylation at S157 and S239 is induced during hypoxia in vitro and during ischemia preconditioning in vivo. The preconditioning-induced VASP phosphorylation inactivates α_IIb_β_3_ integrin receptor on platelets, which results in the reduced formation of organ compromising PNCs, demonstrating that VASP phosphorylation in platelets is a protective mechanism against the deleterious effects of ischemia [142].

## 7. Role of Ena/VASP Proteins in Endothelial Barrier Function

Endothelial cells line vessel walls and form a semi-permeable barrier between the blood and the underlying tissue. Similar to epithelial cells, endothelial barrier function depends on interendothelial junctions, tight junctions (TJ) and adherens junctions (AJ), that are connected to the underlying actin cytoskeleton. In contrast to epithelial cells, however, the endothelial barrier needs to be tightly and dynamically regulated [144,145]. On the one hand the barrier needs to permit the controlled paracellular flux of molecules to meet the physiological requirements of the underlying tissue and the transmigration of immune cells to fight inflammation and infections. On the other hand, the barrier is of utmost importance to restrict uncontrolled leakage of fluid to prevent tissue edema formation. Numerous pathologic conditions are associated with endothelial barrier breakdown, ranging from a mosquito bite to life-threatening diseases, including sepsis and ischemic stroke [144]. While the importance of transmembrane tight junction proteins (claudins, junction-associated molecules and occluding), and adherens junction proteins (VE-cadherin) for endothelial barrier integrity is immediately apparent, the actin cytoskeleton also plays an essential role in regulating the stability of endothelial cell-cell contacts and vascular permeability [145]. Under healthy/resting conditions, the circumferential cortical actin ring stabilizes interendothelial junctions to limit vascular permeability. Following activation, however, inflammatory mediators and vasoactive substances induce the transition of the cortical actin ring into contractile stress fibers, which are thought to destabilize cell-cell junctions and pull opposing plasma membranes apart to increase paracellular permeability (compare Figure 1). The contribution of actin binding proteins for the regulation of vascular permeability has long been underestimated [145], but numerous reports have established a critical role of Ena/VASP proteins in endothelial barrier function in vitro and in vivo [22,37,38,146,147,148,149,150,151,152,153,154,155,156,157,158,159,160,161].

In a differential proteomics screen in endothelial cells, αII-spectrin was identified as novel VASP-binding protein. αII-Spectrin bound directly to the proline-rich region of VASP via its SH3 domain and PKA-mediated phosphorylation of VASP on S157 abrogated the association in vitro and in sparse/migrating cells. In confluent cells, αII-spectrin colocalizes with non-phosphorylated VASP at cell–cell junctions and ectopic expression of the αII-spectrin SH3 domain at cell–cell contacts translocated VASP, initiated cortical actin cytoskeleton formation, and decreased endothelial permeability. Conversely, the permeability of VASP-deficient endothelial cells was increased but barrier function in VASP-deficient cells was restored by its reconstitution. Bradykinin (an inflammatory peptide hormone)-induced edema formation was significantly increased in VASP-deficient mice and VASP-deficiency also increased blood brain barrier damage and edema formation after ischemic stroke [22,37]. Furthermore, VASP binding to αII-spectrin attenuated the caspase-induced cleavage of αII-spectrin in apoptotic endothelial cells [162]. While these reports highlight the importance of VASP for formation of the cortical actin ring, that stabilizes interendothelial junctions, other mechanisms contributing to reduced barrier function in Ena/VASP-deficient endothelial cells have been proposed. Furman and colleagues demonstrated that Ena/VASP triple-deficient mice die in utero and display impaired vascular integrity and edema formation. However, the authors proposed that impaired response to shear stress and altered actomyosin contractility is involved in vascular dysfunction in Ena/VASP-deficient mice [38]. Other proposed mechanisms involve PKA-dependent Rac1 activation [163] and impaired integrin-mediated adhesion to the extracellular matrix in focal adhesions [151,157]. Irrespective of whether different mechanisms exist in parallel, potentially in an organ specific manner, all of the published studies confirmed a crucial role of Ena/VASP proteins in reducing endothelial cell permeability.

## 8. Role of VASP in Leukocyte Infiltration, Polarization, and Vascular Repair after Ischemia

The treatment of ischemic vascular diseases remains a major challenge in cardiovascular medicine [164,165]. Therapeutic intervention to promote reperfusion and function within ischemic tissue represents a promising goal to improve the life of patients. However, this requires a complex interplay of angiogenesis (capillary sprouting from preexisting vasculature) in the ischemic tissue and arteriogenesis (enlargement/remodeling of preexisting collateral arteries into conductance vessels) upstream and around the occlusion in the non-ischemic tissue to reestablish tissue perfusion [166,167]. Due to a lack of understanding of the underlying mechanisms, clinical trials based on the delivery of growth factors through protein or gene transfer have not met the high expectations raised by preclinical studies [165,168]. 

**Figure 7:**
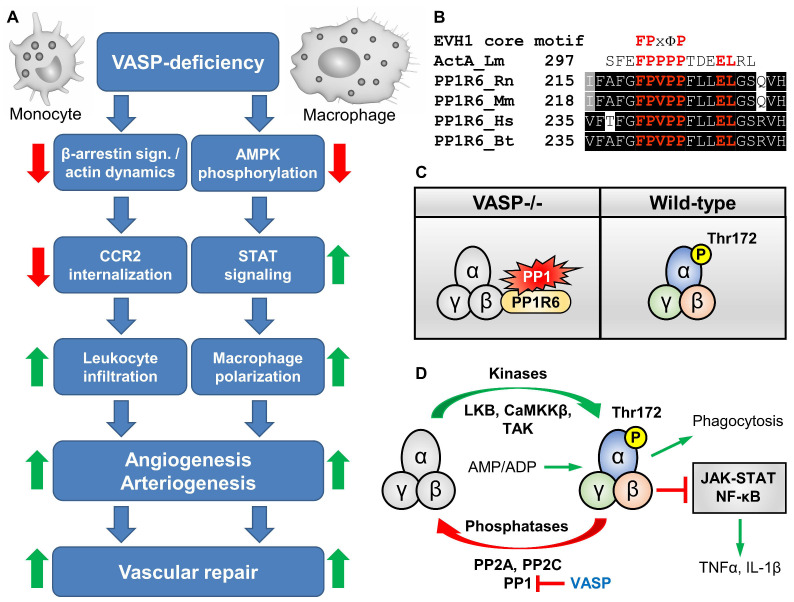
**Proposed model how VASP-deficiency increases monocyte recruitment, macrophage polarization and vascular repair after ischemia.** (**A**) VASP-deficiency drives monocyte infiltration into the ischemic muscle through decreased CCR2 receptor internalization. The latter is likely caused by reduced β-arrestin-2 signaling and/or reduced actin dynamics that drive membrane trafficking. VASP-deficiency impairs AMPK phosphorylation (activation), which drives STAT1-dependent macrophage polarization and CCL2 release. Together, the two mechanisms synergistically increase leukocyte recruitment into the ischemic tissue, which in turn drives angiogenesis, arteriogenesis and vascular repair. (**B**) Sequence alignment of rat (Rn), mouse (Mm), human (Hs), and bull (Bt) PP1-R6 protein sequence. The putative VASP EVH1 motif is highlighted in red. The EVH1 core binding motif and the second, high-affinity EVH1 binding motif of listeria (Lm) ActA are shown for comparison (x, any amino acid; Φ, hydrophobic amino acid). (**C**,**D**) Proposed model how VASP deficiency impairs AMPK phosphorylation in macrophages. Phosphorylation of AMPK at Thr-172 is induced by AMP/ADP and the upstream protein kinases LKB1, CaMKKβ and TAK1. Dephosphorylation of AMPK at Thr172 is regulated by protein phosphatases PP1, PP2A and PP2C. (**C**) In wild-type cells, VASP-binding to PP1-R6/PP1 complex limits the PP1-dependent de-phosphorylation of AMPK. In the absence of VASP, AMPK dephosphorylation by the PP1-R6/PP1 complex is increased. (**D**) In macrophages, AMPK activation drives phagocytosis and inhibits JAK-STAT and NF-κB signaling, thereby limiting the expression of pro-inflammatory cytokines including TNFα and IL-1β. Conversely, impaired AMPK activity in VASP-deficient macrophages increases the STAT1-mediated pro-inflammatory phenotype and limits phagocytosis. Figure modified from [63].

Given the role of VASP in endothelial and smooth muscle cells, a beneficial function of the protein in vascular repair and tissue reperfusion was hypothesized. Surprisingly, however, vascular repair and blood flow recovery were increased after ischemia in VASP-deficient mice. This unexpected effect was not directly endothelium dependent and attributed to the function of VASP in leukocyte infiltration and polarization, and chemokine receptor trafficking [25]. In fact, leukocyte attraction and polarization play an important role in arteriogenesis, angiogenesis, and regeneration of the ischemic tissue [169,170]. VASP-deficiency significantly increased the CCL2 chemokine release from macrophages while at the same time CCR2 internalization in VASP^−/−^ monocytes and neutrophils was impaired, thereby synergistically increasing leukocyte recruitment into the inflamed tissue and tissue repair. Conversely, arteriogenesis after experimental arterial occlusion is impaired in mice lacking the chemokine receptor CCR2 [169]. VASP-deficiency also increased macrophage polarization through increased signal transducer and activator of transcription (STAT)1 expression, which augmented the release of chemokines, cytokines, and growth factors to promote leukocyte recruitment and vascular repair [25] (Figure 7A). 

How VASP regulates STAT1 signaling was not known. Given that AMPK is an important regulator of macrophage polarization and AMPK activation inhibits STAT1 expression [171,172,173,174], the role of AMPK in determining the phenotype of VASP-deficient macrophages was investigated. While a basal activity of AMPK (phosphorylation on Thr172) was detected in macrophages from wild-type mice, AMPK phosphorylation was significantly reduced in VASP-deficient M1 macrophages in vitro and the expression of the pro-inflammatory cytokines TNFα and IL-1β was increased in these cells [63]. A link between VASP and AMPK phosphorylation in macrophages has not been reported to date. However, absence of VASP has previously been shown to reduce AMPK activation and fatty acid oxidation in hepatocytes. Interestingly, restoring AMPK activity by AICAR treatment in vivo rescued the liver phenotype in VASP-deficient mice [175].

Given that AMPK activation enhances the phagocytic capacity of macrophages and neutrophils [176,177,178], the phagocytic capacity of VASP-deficient macrophages was assessed. VASP^−/−^ macrophages displayed significantly reduced phagocytic capacity compared to wild-type controls. Interestingly, activation of AMPK with berberine increased phagocytosis in VASP-deficient cells to a level that was statistically indistinguishable from wild-type macrophages [63]. Thus, impaired AMPK activity in VASP^−/−^ macrophages may, at least in part, explain the decreased phagocytosis capacity. While other investigators have also reported a reduced phagocytic capacity of VASP-deficient cells, this phenomenon was attributed to a direct effect of VASP on the phagocytosis-induced reorganization of actin filaments in the phagocytic cup and AMPK activity was not investigated in these cells [128,179].

Phosphorylation of AMPK on Thr-172 is dynamically regulated by several kinases and phosphatases [180]. Mechanistically, VASP bound directly to protein phosphatase 1 (PP1) regulatory subunit 6 (PP1-R6) [63], which recruits PP1 to dephosphorylate and thereby inactivate AMPK [181]. Notably, PP1-R6 contains a conserved peptide motif (Figure 7B) that closely resembles the high affinity EVH1 binding motif of ActA [47]. Given that VASP is also dephosphorylated by PP1 [182], it is tempting to speculate that loss of VASP increases the PP1-R6 targeting of AMPK and thus the dephosphorylation of the kinase (Figure 7C). Since VASP is phosphorylated by AMPK [103], this may constitute a negative feedback loop that attenuates AMPK activation in VASP-deficient macrophages (and hepatocytes), which in turn drives a STAT1-mediated pro-inflammatory phenotype and at the same time impairs phagocytosis (Figure 7D).

Other studies have corroborated important functions of Ena/VASP proteins in myeloid cells, including the impaired phagocytosis in macrophage-like cells transfected with GFP-ActA repeats and impaired macropinocytosis, spreading and migration of EVL/VASP-double deficient dendritic cells [128,183]. However, there is also evidence that the proteins are crucial for lymphocyte functions. For example, VASP and EVL have been shown to regulate the trafficking of activated T cells by promoting diapedesis during transendothelial migration. In contrast to the findings in VASP-deficient monocytes and neutrophils, however, T-cell diapedesis and trafficking to inflamed skin was impaired in EVL/VASP-deficient cells [184]. Furthermore, Ena/VASP mediated actin polymerization contributes to naïve CD8+ T-cell activation and expansion by promoting T-cell—antigen presenting cell interactions in vivo [185]. In natural killer cells, effector lymphocytes of the innate immune system, VASP-dependent actin polymerization was required for maintaining lytic granule convergence during natural killer cell-mediated killing [186].

## 9. Role of Ena/VASP Proteins in the Mammalian Heart

In the mammalian heart, the α-cardiac actin isoform is the major constituent of sarcomere thin filaments. However, circumstantial evidence suggested the existence of a different actin isoform at Z- and intercalated discs, possibly β- or γ-cytoplasmic actin [187,188]. Cardiac VASP and Mena expression was upregulated in neonatal and hypertrophic hearts, conditions in which the heart is confronted with an increased mechanical workload. Left-ventricular performance was increasingly impaired in adult VASP-, Mena-, and Mena/VASP double deficient mice but only the double-deficient animals developed dilated cardiomyopathy [12]. Thus, it seems that Mena and VASP can compensate for the loss of each other to a certain extent. In contrast, intra-atrial and intraventricular propagation of electrical signals was equally delayed in Mena/VASP single- and double-deficient mice in the heart. Mena and VASP specifically interacted with a splice variant of αII-spectrin (SH3i), which is exclusively localized at Z- and intercalated discs of cardiomyocytes. At the latter sites, Mena localized to the edges of the sarcomeres, where the thin filaments are anchored. Importantly, actin filaments at these sites are composed of the β-cytoplasmic actin isoform. In contrast, colocalization of Mena with α-cardiac or γ-cytoplasmic actin fibers was not detected, indicating, that Ena/VASP proteins specifically regulates β-cytoplasmic actin networks. Ena/VASP proteins readily polymerize actin prepared from rabbit skeletal muscle in vitro [11,18,31], indicating that the proteins themselves do not distinguish between the different actin isoforms. However, profilin can discriminate between muscle and cytoplasmic isoforms of actin [189] and may therefore direct Ena/VASP proteins towards cytoplasmic actin networks. However, targeting of Ena/VASP proteins to β-cytoplasmic networks by EVH1- or SH3-domain mediated interactions, including αII-spectrin, vinculin, and zyxin is also possible. Interestingly, gene deletion of the latter proteins is also associated with compromised heart development and function [190,191,192]. In Mena/VASP double deficient mice, Z-discs appeared wavy and fractured and actin filaments of the I-band appeared disorganized [12]. Mena/VASP double-deficiency also impaired the structural integrity of intercalated discs and especially the morphology of gap junctions was markedly impaired, thereby providing a structural reason for the disturbed propagation of electrical signals.

Several other studies have investigated the role of Ena/VASP proteins in the mammalian heart. Again, VASP was reported to be upregulated in hypertrophied hearts [193]. Analysis of Mena-deficient animals revealed impaired cardiac contraction and delayed conduction of electrical impulses, resulting from the malformation of intercalated discs and gap junction assembly [194]. Instead of using single or combined gene deletion, Eigenthaler and colleagues used a dominant negative approach and expressed the VASP-EVH1 domain specifically in cardiomyocytes. Transgenic mice showed displacement of both VASP and Mena from intercalated disks and developed dilated cardiomyopathy and myocyte hypertrophy. However, mice with high levels of transgene expression displayed severe bradycardia and died early in postnatal development [195], which contrasts with other observations [12]. Currently, it’s unclear, if these findings originate from side effects of the transgenic protein expression or if the approach also disrupted a potential function of cardiac EVL. To date, cardiac EVL expression or function has not been systematically analyzed.

## 10. Role of VASP in Supporting the Conducted Vasodilation along the Vessel Wall

Cellular coupling and signal transmission via gap junctions is of functional importance in vascular tissue. Endothelial and smooth muscle cells in the vessel wall are coupled heterocellularly via myoendothelial gap junctions providing a signaling pathway in the transversal direction, i.e., from endothelial to smooth muscle cells, thereby at least partially translating endothelial cell hyperpolarization into smooth muscle cell relaxation (reviewed in [196,197,198]). In addition, endothelial cells and vascular smooth muscle cells are homocellularly coupled, thereby providing a longitudinal signaling pathway along the vessel wall. The latter serves to coordinate vascular tone in arterioles in the microcirculation by integrating cells into a tightly coupled syncytium [199]. It was suggested that this pathway contributes to the matching of oxygen supply and tissue needs [200]. In fact, under conditions of enhanced tissue oxygen needs (skeletal muscle contraction), mice with impaired gap junctional communication exhibit reduced dilations indicating the physiological importance of this pathway in active hyperemic responses induced by metabolic demand [201].

**Figure 8:**
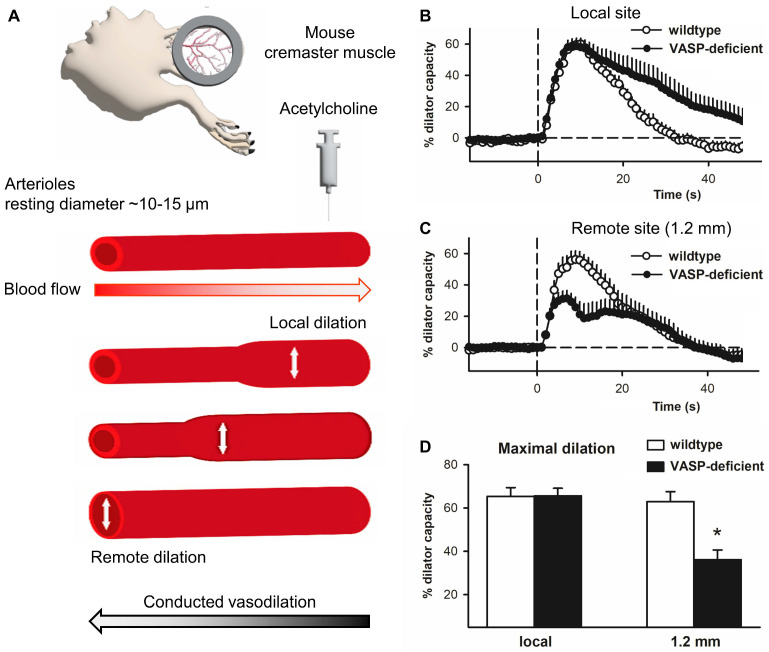
**Critical role of VASP in supporting the conducted vasodilation along the vessel wall.** (**A**) Schematic diagram showing how conducted vasodilations can be studied in the microcirculation in vivo. A glass micropipette is positioned in close proximity to a second- or third-order arteriole (resting diameter ~10–15 μm) of the cremaster muscle. Locally confined application of acetylcholine is used to elicit endothelial hyperpolarization, while observing the local and the remote/upstream vasodilation. (**B**–**D**) Conducted vasodilations in VASP-deficient vs. wild-type mice. Arteriolar diameter changes are plotted as % of dilator capacity over time at the local stimulation site (**B**) and upstream, remote sites at a distance 1.2 mm (**C**). The stimulation with acetylcholine (at time point 0) induced a rapid dilation with similar maximal ampitude at the local site in wild-type (white symbols) and VASP-deficient mice (black symbols) (**B**,**D**). While the dilatory amplitude did not decrease up to a distance of 1.2 mm in wild-type mice, the amplitude of the dilation was significantly attenuated at the remote site in VASP-deficient mice (**C**,**D**). * indicates *p* < 0.05 vs. local dilation (paired *t*-test, Bonferroni corrected). Figure modified from [23].

The longitudinal conduction pathway is mainly provided by endothelial cells, which are tightly coupled through gap junctions composed of connexin 37 and 40. Of these connexins, connexin 40 is of the utmost importance and deletion of connexin 40 impairs longitudinal signaling [202]. Such tight coupling is required for the spread of locally induced vasodilations along the vessel wall—a phenomenon referred to as conducted vasodilation [203]. Conducted vasodilation can be studied in vivo by locally confined application of substances that induce an endothelial hyperpolarization, such as acetylcholine, while observing the local stimulation site and, subsequently, the upstream remote sites of the arteriole [202,204] (Figure 8A). In this manner, conducted vasodilation was studied in arterioles of cremaster muscle in wild-type and VASP^−/−^ mice [23].

Acetylcholine induced a short dilation at the local site that was also observed at remote, upstream sites without an attenuation of the amplitude (wild-type; Figure 8B–D). The local dilation was of a similar amplitude in VASP-deficient mice (Figure 8B) and also conducted to remote sites without delay. However, in contrast to wild-type animals, the amplitude of the dilation was significantly reduced in VASP^−/−^ mice at the remote site [23] (Figure 8C,D). Such attenuated conducted vasodilation resembles the findings in mice lacking connexin 40 in endothelial cells [202,204]. Similar to the findings in the mammalian heart, this suggests that VASP is important for endothelial gap junction assembly in the vessel wall. Consistently, VASP colocalized with connexin 40 at cell-cell contacts of human endothelial cells (Figure 2E). Taken together, the data suggest that VASP exerts a critical role in supporting the spread of hyperpolarization along the endothelial cell layer and consequently in the conducted vasodilation along the vessel wall.

## 11. Role of Mena and VASP in Smooth Muscle Cell Relaxation

**Figure 9:**
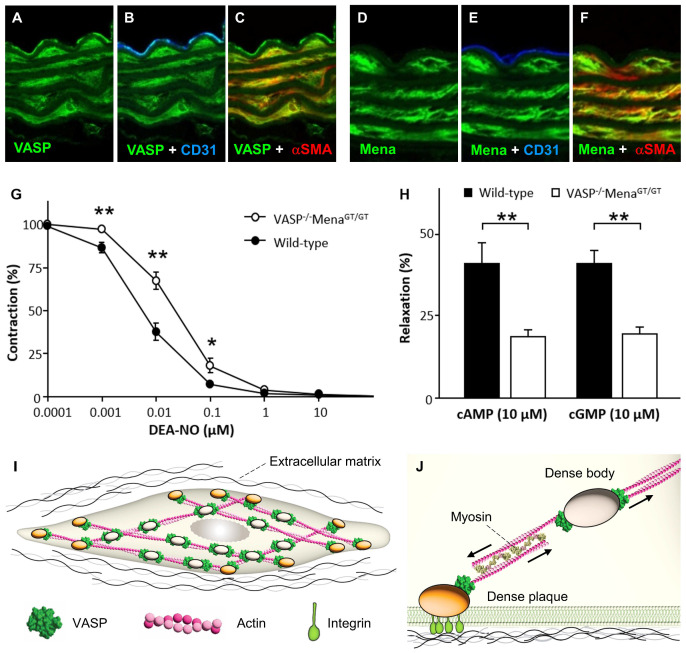
**Role of Mena and VASP in smooth muscle cell relaxation.** Confocal microscopy to investigate the expression of VASP (**A**–**C**) and Mena (**D**–**F**) in endothelial cells and smooth muscle cells of the wild-type mouse aorta. Staining with CD31 and α-smooth muscle actin specific antibodies was used to identify endothelial cells and smooth muscle cells, respectively. (**G**,**H**) Myograph experiments with aortic rings from VASP^−/−^Mena^GT/GT^ or wild-type mice. Significantly impaired smooth muscle cell relaxation was observed in rings form VASP^−/−^Mena^GT/GT^ mice in response to increasing concentrations of the NO-donor DEA-NO or in response to 10 µM of the cAMP- or cGMP-analogs, Sp-5-6-DCI-BIMPS and 8-Br-pCPT-cGMP, respectively; * *p* < 0.05, ** *p* < 0.01. (**I**) Top view of a vascular smooth muscle cell, with dense plaques in orange and dense bodies in grey. VASP (green) is associated with actin fibers (magenta) at the dense plaques and dense bodies. (**J**) Side view of dense plaques and dense bodies, where actin filaments are anchored to the extracellular matrix and within the cytosol, respectively. Actomyosin contraction forces are indicated by arrows. Figure modified from [135].

VASP is strongly expressed in endothelial cells and vascular smooth muscle cells [136] (Figure 9A–C)) and a prominent target of PKA and PKG. Given that cyclic nucleotide signaling pathways are crucial for smooth muscle cell relaxation and vasodilation, it was speculated that VASP and VASP phosphorylation would be an important mediator in these processes. Surprisingly, cAMP- and cGMP-induced relaxation of VASP-deficient vascular smooth muscle was undistinguishable from wild-type controls and functional compensation by other Ena/VASP proteins was suggested to account for the effect [130]. 

However, expression of EVL in vessel walls is elusive and it is controversial whether Mena is expressed in vascular smooth muscle cells at all. While Gambaryan and co-workers found strong Mena expression in the blood vessel wall [136], others claim that VASP is the only Ena/VASP protein family member expressed in the aorta [205].

A recent study analyzed the role of Ena/VASP proteins for smooth muscle relaxation in more detail and investigated a potential functional redundancy of the proteins [135]. In contrast to a previous report [130], VASP deficiency was found to significantly impair the acetylcholine (ACh)- and NO-induced relaxation of the aorta ex vivo. However, this effect was age-dependent and only observed in aortic rings from 7-month-old animals, but not in rings from 3-month-old mice. Interestingly, ACh- and NO-dependent relaxation was already significantly impaired in mesenteric artery rings from 4-month-old VASP^−/−^ mice, indicating that smaller arteries are more susceptible to VASP-deficiency [135]. In fact, a recent study has revealed that the NO-induced dilation of arterioles in the microcirculation of VASP-deficient mice was also significantly impaired [23]. Interestingly, the cGMP-dependent smooth muscle relaxation was not impaired in VASP^−/−^ vessels, suggesting that the expression and/or activity of the NO-sensitive soluble guanylyl cyclase may be altered in the absence of VASP.

Unlike VASP, EVL was not detectable in the aorta and EVL-deficiency had no impact on its agonist-induced relaxation [135]. This indicates a more specialized function of EVL in different vascular beds. Indeed, EVL was previously implicated in endothelial barrier function and sprouting angiogenesis [120,150], indicating a function of EVL in small vessels and capillaries rather than in the aorta. Notably, EVL lacks the preferred PKG-dependent phosphorylation site, which is conserved in VASP and Mena (Figure 3), further indicating that EVL may not be involved in NO/cGMP-mediated vessel relaxation. Nevertheless, PKG may also phosphorylate the preferred PKA phosphorylation site (S157 in human VASP) and additional studies in smaller arteries and arterioles are required to fully clarify the role of EVL in vascular smooth muscle relaxation.

Mena promoter activity and protein expression was high in vascular smooth muscle cells, but hardly detectable in endothelial cells of the aorta [135] (Figure 9D–F). In vascular reactivity studies with aortic rings from VASP^−/−^Mena^GT/GT^ mice, which lack VASP and display only minimal vascular Mena protein [12], NO-mediated relaxation was more severely impaired than that of tissues from VASP^−/−^ mice. Moreover, cAMP- and cGMP-induced relaxations were also significantly impaired in aortic rings of VASP^−/−^Mena^GT/GT^ mice, which was not the case for VASP^−/−^ mice [135] (Figure 9G,H).

The mechanisms underlying Mena/VASP-mediated smooth muscle cell relaxation are currently elusive. However, the molecular interplay is likely related to actin cytoskeletal dynamics, rather than alterations in cytosolic calcium concentrations and/or the sensitivity of the contractile apparatus towards calcium ions. Actin and actin binding proteins are now known to regulate the development of mechanical tension in smooth muscle cells [206,207] and the functional role of actin polymerization during contraction is likely independent of the calcium triggered actomyosin crossbridge cycle [208]. Contractile stimuli are thought to initiate the cortical actin polymerization and the assembly of cytoskeletal/extracellular matrix adhesions, which strengthen the membrane for the transmission of force generated by the contractile machinery [206,207,208]. Conversely, inhibition of actin polymerization may serve to relax smooth muscle tissue. In vascular smooth muscle cells, VASP is localized to dense plaques and dense bodies [209] (Figure 9I and J), where actin filaments are anchored to the extracellular matrix and within the cytosol, respectively [208]. In large arteries, the α-smooth muscle actin isoform is the major constituent of thin filaments, but β- and γ-cytoplasmic actin and γ-smooth muscle actin isoforms are also present. Interestingly, β-cytoplasmic actin is associated with dense plaques and dense bodies [206]. Similar to the situation in cardiomyocytes, this suggests that VASP is a selective regulator of β-cytoplasmic actin dynamics in vascular smooth muscle cells. Several studies have indicated a role of VASP and VASP phosphorylation in regulating actin polymerization and contraction in smooth muscle cells. VASP was shown to co-localize with hot spots of actin polymerization at the cell cortex of vascular smooth muscle cells and VASP phosphorylation, known to inhibit actin polymerization, was decreased after stimulation with phenylephrine [205]. In airway smooth muscle cells, acetylcholine triggered the PKC-mediated phosphorylation of VASP at S157 and the formation of VASP-vinculin complexes at membrane adhesion sites, which is necessary for VASP-mediated actin polymerization and tension generation. Although forskolin, which induces cAMP/PKA signaling, also induced VASP Ser157 phosphorylation and membrane localization, it did not stimulate actin polymerization. This may be related to the fact that forskolin also induced phosphorylation of S239 [210], which is known to inhibit actin polymerization [18]. This may constitute a potential mechanism for cAMP/PKA- and cGMP/PKG-induced smooth muscle relaxation mediated by VASP (and Mena). Consistently, smooth muscle cell-induced contraction of collagen gel was reduced in cells with wild-type VASP after NO stimulation and when VASP-deficient cells were reconstituted with a phosphomimetic S239D-VASP mutant [211].

## 12. Interaction of VASP and AKAP12 in VEGF-Induced Endothelial Cell Migration and Sprouting

A-kinase anchoring proteins (AKAPs) recruit PKA to specific subcellular loci, thereby providing discrete spatiotemporal control of downstream phosphorylation events [212]. Surprisingly, little is known about the spatiotemporal regulation of Ena/VASP through AKAPs. However, a recent study investigated the interaction of VASP and AKAP12 during VEGF-induced endothelial cell migration and spouting. In migrating endothelial cells, AKAP12 was detected at the leading edge of lamellipodia, where it colocalized with actin filaments and VASP. AKAP12^−/−^ retinas displayed a significantly delayed radial sprouting of the vascular plexus over the first postnatal week, indicating that the lack of AKAP12 attenuates endothelial cell migration [213]. Mass spectrometry-based proteomics revealed association of AKAP12 with multiple key regulators of actin filament-based movement, including VASP and components of the Arp2/3 complex, and confirmed VASP/AKAP12/PKA complex formation. Consistently, VEGF-stimulated phosphorylation of VASP by PKA was dependent on AKAP12 and AKAP12 co-localized with phospho-S157-VASP at the leading edge of migrating endothelial cells. The results suggest that compartmentalized AKAP12/PKA signaling mediates VASP phosphorylation at the leading edge of migrating endothelial cells to translate angiogenic stimuli into altered actin dynamics and cell movement [213]. Given that the preferred PKA phosphorylation site of VASP is functionally conserved in the two other Ena/VASP family members, these findings may also apply to EVL and Mena. Indeed and similar to AKAP12, endothelial-specific deletion of EVL compromised the VEGF-induced radial sprouting of the vascular plexus in the postnatal murine retina [120]. However, the importance of AKAP-mediated VASP phosphorylation in endothelial cell migration seems not to be limited to the VEGF pathway. In fact, a later study showed that promotion of PDGF-induced endothelial cell migration by phosphorylated VASP also depends on PKA anchoring via AKAP [214]. Given that both AKAP12 and Ena/VASP proteins regulate endothelial barrier function [22,38,215,216], it is tempting to speculate that the AKAP12-Ena/VASP complex formation may also regulate PKA-regulated vascular permeability. There is at least circumstantial evidence to support this hypothesis as stabilization of endothelial barrier function by cAMP-mediated Rac 1 activation was dependent on AKAP-mediated PKA anchoring and VASP [155].

## 13. EVL Regulates VEGF Receptor 2 Internalization and Signaling in Developmental Angiogenesis

**Figure 10:**
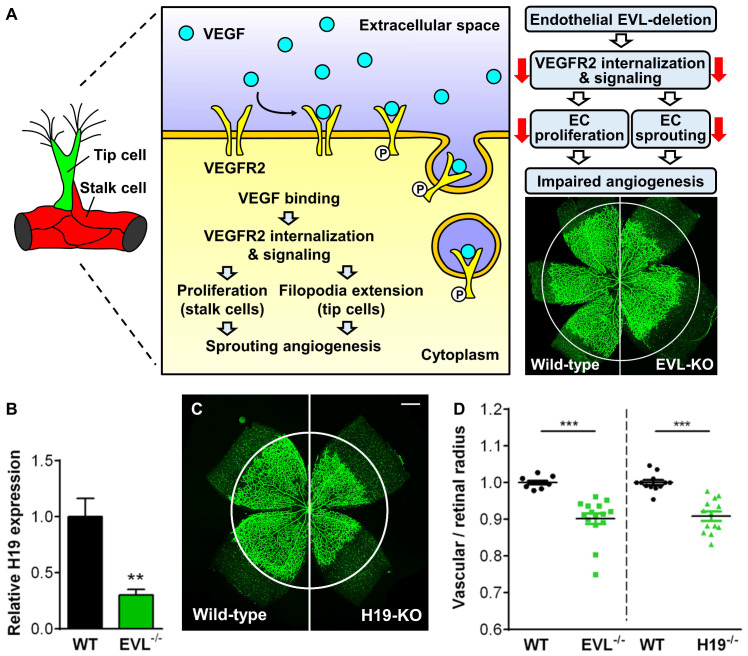
**EVL deficiency impairs developmental angiogenesis in the postnatal retina.** (**A**) Graphical summary how EVL regulates developmental angiogenesis in the postnatal retina. Genetic deletion of EVL in endothelial cells impairs VEGF receptor-2 internalization and signaling. This decreases VEGF-induced endothelial stalk cell proliferation, tip cell density and filopodia formation, which culminates in an impaired sprouting angiogenesis in the postnatal retina. (**B**–**D**) Reduced expression of lncRNA H19 contributes to the impaired retinal angiogenesis in EVL-deficient mice. (**B**) RNA levels of lncRNA H19 in wild-type and EVL^−/−^ retinal endothelial cells at postnatal day 5; ** *p* < 0.01. (**C**,**D**) Delayed postnatal retinal angiogenesis in H19-deficient mice. (**C**) Retinas of wild-type and H19^−/−^ mice were harvested on postnatal day 5, the vasculature was stained with Isolectin B4 and analyzed by confocal microscopy. Comparison of one representative retina from wild-type and H19-deficient animals, each. Scale bar: 200 μm. (**D**) Statistical analysis of the radial outgrowth of wild-type and global H19-deficient animals normalized to the retinal radius as well as littermate controls; radial outgrowth of EVL-deficient mice is shown for comparison; *** *p* < 0.001. Figure modified from [220].

During angiogenesis, highly motile and invasive endothelial tip cells form actin-rich lamellipodia and filopodia, which probe the environment for guidance cues, such as VEGF, and thereby determine the direction of growth [217]. VEGF binding triggers phosphorylation of endothelial VEGF receptor 2 (VEGFR2), which is crucial for the activation of downstream signaling targets, including ERK1/2, that control proliferation, migration, and sprouting [218,219]. Although the surface expression of VEGFR2 is a prerequisite for ligand binding, the endocytosis of the receptor is essential to activate many, if not all of the downstream signaling pathways, including ERK1/2 [218] (Figure 10A). 

Despite their pivotal role in tip cell navigation, little is known about the processes regulating filopodia assembly in endothelial cells [20,217,221]. However, endothelial tip cells share many structural and molecular similarities with axonal growth cones, which also probe the microenvironment to translate guidance cues into directed cell migration [221,222]. Given the established role of Ena/VASP proteins for filopodia formation in axonal growth cones [30,68], the role of Ena/VASP proteins was investigated in developmental angiogenesis [120]. While postnatal retinal endothelial cells expressed VASP and EVL, Mena was hardly detectable. In contrast to VASP, global and endothelial-specific EVL deletion resulted in a significantly delayed radial sprouting of the vascular plexus in postnatal retina. However, this does not rule out a role for VASP in angiogenesis. Indeed, ANP-mediated VASP phosphorylation has been implicated in regulation of angiogenesis, at least in vitro [223]. Endothelial-specific EVL deletion was also associated with a significant reduction in tip cell and filopodia numbers at the vascular front and a significant decrease in endothelial cell proliferation. This was linked to impaired VEGF signaling in EVL-deficient endothelial cells. Indeed, VEGF failed to increase the proliferation of EVL-deficient cells and VEGF-induced endothelial cell sprouting was completely blunted in EVL-deficient aortic rings and endothelial cells. Mechanistically, VEGFR2 internalization and phosphorylation were significantly impaired in VEGF-stimulated EVL^−/−^ cells, which translated into reduced ERK1/2 phosphorylation both in vitro and in vivo [120] (Figure 10A).

In addition to altered internalization of growth factor receptors, the Ena/VASP proteins may alter angiogenesis by other mechanisms. For example, a recent study reported that the expression of the long non-coding RNA H19 was significantly reduced in retinal endothelial cells from postnatal EVL^−/−^ mice and in siRNA-transfected human endothelial cells under hypoxic conditions [220] (Figure 10B). Similar to EVL^−/−^ mice, H19-deficient animals [224] displayed a significantly impaired radial sprouting of the vascular plexus on postnatal day 5 (Figure 10C,D), indicating that down-regulation of the lncRNA in EVL-deficient mice may contribute to the impaired angiogenic sprouting [220]. Consistent with this finding, knockdown of H19 in glioma-associated endothelial cells suppressed glioma induced angiogenesis [225]. Interestingly, H19 was recently shown to promote VEGF expression and bioavailability via Esm1 [226,227] and hypoxia inducible factor 1α (HIF-1α) [228]. Together, the data suggest that loss of EVL not only impairs VEGFR2 internalization and downstream signaling, but also impairs VEGF expression and bioavailability in the hypoxic retina via downregulation of lncRNA H19.

## 14. Role of Ena/VASP Proteins in Endocytosis and Receptor Trafficking

While many studies have focused on deciphering the role of Ena/VASP proteins in the formation of lamellipodia, microspikes and filopodia protrusions during cell migration [19], it is becoming increasingly clear that the proteins are also crucial regulators of membrane and receptor trafficking. Ena/VASP-dependent receptor internalization/trafficking seems to occur predominantly in the context of chemotaxis and guidance cue mediated cell migration. This includes leukocyte chemotaxis in response to CCR2 trafficking [25], breast cancer cell invasion and metastasis in response to EGFR internalization [15,26], VEGFR2 internalization and signaling in developmental angiogenesis [120], ephrin/Eph mediated fibroblast repulsion via Eph receptor internalization [126], and potentially other situations, in which Ena/VASP proteins were implicated in attractive or repulsive guidance cue signaling [30,59,60,229,230]. However, there is also evidence that VASP is involved in receptor recycling to the membrane, as VASP regulates the Rab11-dependent plasma membrane targeting of TGF-β receptors [231]. How exactly Ena/VASP regulates these apparently opposing processes and which receptor classes and subclasses are affected, potentially in a cell type–specific manner, is currently not fully understood. For example, EVL and Mena were shown to regulate internalization and signaling of the receptor tyrosine kinases VEGFR2 and EGFR, respectively, whereas VASP was implicated in endocytosis of the G-protein coupled receptor CCR2 and TGF-β receptor II [25,26,120,231].

The impact of Ena/VASP proteins on receptor cycling is likely to involve direct or indirect interaction with the respective receptors or receptor complexes and/or the regulation of actin dynamics that drive membrane trafficking. Supporting the former concept is that Ena/VASP proteins have been shown to directly interact with several guidance cue receptors, including Robo, Sema6A and Dlar [59,60,230]. Furthermore, VASP forms complexes with the chemokine receptor CCR2 and β-arrestin-2 in leukocytes [25] and TGF-β receptor II in hepatic stellate cells [231]. Given that CCR2 follows the canonical G protein–coupled receptor (GPCR) trafficking pathway, which requires interaction with arrestins to target the receptor for internalization [232], this may contribute to the impaired internalization of CCR2 in VASP-deficient leukocytes. Ena/VASP association with receptor complexes may also be mediated by binding to adapter proteins, such as the Nck family [127,128]), which are known to connect receptor (and non-receptor) tyrosine kinases to the machinery of actin reorganization, thereby regulating signal transduction of VEGF- and Eph-receptors and others [233,234]. The lack of Ena/VASP complex formation with Nck family proteins may therefore contribute to the impaired Eph/VEGFR2 receptor internalization observed by blocking or genetic deletion of Ena/VASP proteins [120,126]. 

Regulation of actin dynamics has a central role in processes that reshape the plasma membrane. This is not limited to protrusions of lamellipodia and filopodia during cell migration but also includes different forms of endocytosis, including phagocytosis, macropinocytosis and clathrin- or caveolae-mediated endocytosis [1,5]. Actin polymerization at developing endocytic pits shares many aspects with lamellipodia formation in migrating cells, which is dynamically regulated by Ena/VASP proteins [1] (compare Figure 1). Furthermore, actin assembly in endosomal “comet tails” seems to push the endosomes along. This is reminiscent of the intracellular bacterial pathogen Listeria, which uses a very similar actin rocketing mechanism for its motility inside mammalian host cells [1]. This is worth mentioning as the Listeria surface protein ActA has been shown to recruit Ena/VASP proteins to promote local actin polymerization and intracellular motility [1,27,47]. A recent study has confirmed this in the professional phagocyte Dictyostelium, which only expresses VASP but not Mena or EVL [179]. During Dictyostelium phagocytosis of yeast particles, lamellipodia-like actin protrusions propagated over the surface of the yeast particles and VASP localized to the very tip of the protruding cup, i.e., in vicinity of the actively elongating actin filaments. After cup closure, VASP condensed at the distal side of internalized phagosomes and initiated localized de-novo actin assembly to propel the phagosome by an actin-rich comet tail deeper into the cell. In line with the role of Ena/VASP proteins in Listeria movement, travelled distance and speed of rocketing phagosomes in VASP-deficient Dictyostelium cells were markedly impaired [179]. Similar to the results in VASP^−/−^ macrophages [63], loss of VASP in Dictyostelium resulted in substantial defects in phagocytosis and macropinocytosis. Given that VEGF-induced internalization and signaling of VEGFR2 is largely mediated by macropinocytosis [235], these findings also provide a rational for the impaired VEGFR2 internalization in EVL-deficient endothelial cells [120].

Last but not least, VASP has been shown to form complexes with Rab11, a key regulator of recycling endosomes, in hepatic stellate cells, thereby regulating the TGF-β receptor II recycling to the plasma membrane [231]. While this provides yet another potential mechanism for Ena/VASP-dependent receptor trafficking, it remains to be determined if this constitutes a common role to all Ena/VASP family members. Given that actin dynamics are also vital for several steps of exocytosis, including vesicle transport to and attachment at the cell cortex during the pre-fusion phase [236], it will be interesting to see, whether or not Ena/VASP proteins also participate in this aspect of membrane trafficking.

## 15. Redundant and Non-Redundant Functions of Ena/VASP Proteins

The rather mild phenotypes of mutant mice with single Ena/VASP gene deletion were surprising and based on the very similar domain organization of the proteins, functional and mutual compensation of the proteins was hypothesized. There are indeed several lines of evidence that argue for a functional redundancy of the proteins. In mice, Ena/VASP expression patterns differ significantly between the individual family members. Mena and VASP are both expressed in heart, lung, blood vessels, and smooth muscle cells of stomach and intestine. However, VASP is highly expressed in platelets and spleen, whereas Mena has not yet been detected in these tissues. Conversely, Mena but not VASP is abundantly expressed in brain tissue [12,131,135,136]. Consistent with a functional compensation of the Ena/VASP proteins, morphological or functional abnormalities of Mena^−/−^ or VASP^−/−^ mice are mostly found in tissues that predominantly express one family member, i.e., impaired regulation of platelet aggregation in VASP^−/−^ mice and defective brain architecture in Mena null mice [130,132]. In contrast, Mena/VASP double-deficient mice display a much more severe phenotype and die perinatally [134].

Additional evidence for a functional redundancy of Ena/VASP proteins stems from genetic studies in Drosophila. Enabled (Ena) is the only member of the Ena/VASP protein family in Drosophila and Ena^−/−^ mutants are lethal. Genetic, biochemical, and cell biological approaches demonstrated that human VASP can at least partially substitute for a loss of Ena and transgenic VASP expression in the developing Drosophila embryo rescued the lethality of Ena^−/−^ mutants [237]. Functional redundancy has also been revealed in the context of endothelial barrier function [22,38], heart contraction [12], smooth muscle relaxation [135], and actin assembly at cell-cell contacts [35]. However, even in tissues with overlapping expression patterns, non-redundant functions of individual Ena/VASP family members have been observed. For example, loss of EVL in retinal angiogenesis could not be compensated by VASP, although the latter was clearly detected in the postnatal retina as well [120]. Similarly, loss of individual Ena/VASP proteins resulted in significantly impaired filopodia and lamellipodia formation, which could not be compensated by the other family members [238]. This may be explained by differences in binding partners or phosphorylation patterns, or intrinsic differences of the proteins. Consistent with the latter, EVL has been shown to be more efficient than Mena or VASP in generating cell matrix adhesions and traction forces during cell spreading [27,239]. Nevertheless, much remains to be learned to better understand the redundant and unique functions of Ena/VASP proteins, particularly in a cell type and process specific context.

## 16. Ena/VASP Proteins as Therapeutic Targets

Ena/VASP proteins have been linked to various human pathologies, including thrombotic diseases and cancer [69,130,133,240]. As outlined above, VASP-deficiency increases platelet adhesion and aggregation. Furthermore, the phosphorylation status of VASP is frequently used to assess PKA/PKG-dependent platelet reactivity and VASP phosphorylation reduces the detrimental formation of intravascular platelet-neutrophil complexes within ischemic myocardial tissue. Given that many cardiovascular diseases, including heart failure, diabetes and hypertension, are characterized by platelet hyperactivation and impaired cyclic nucleotide signaling [241,242,243,244], it seems logic to increase VASP expression and/or phosphorylation by pharmacologic intervention to modulate platelet reactivity. While increasing VASP expression in platelets is currently not pursued in a clinical setting, it is noteworthy that several antiplatelet therapies, including the P2Y_12_ antagonist Clopidogrel, increase cAMP/PKA signaling and VASP phosphorylation in human platelets [245,246]. Nevertheless, VASP is certainly not the only PKA target downstream of Clopidogrel-mediated P2Y_12_ inhibition.

The regulation of the actin cytoskeleton plays a crucial role in cancer development and progression. Oncogenic cells utilize their intrinsic migratory capacity to invade adjacent tissues and blood vessels, and finally to metastasize [247,248]. Therefore, it is not surprising that actin-binding proteins that functionally link migratory signals to actin remodeling are upregulated in invasive and metastatic cancer cells [247,249]. Indeed, all Ena/VASP family members were found upregulated in different cancers, including breast, cervical, colorectal, pancreatic and lung cancers, and relative expression levels of the proteins have been positively correlated with a poor prognosis and metastasis in patients [250,251,252,253,254,255]. Conversely, Ena/VASP knockdown or deficiency inhibited cancer cell migration and invasiveness in vitro and in vivo [251,253,256]. While knockdown of Ena/VASP proteins in tumors seems challenging in a clinical setting, a recent study has used cell-membrane permeable low molecular weight compounds to disrupt the EVH1-domain mediated interactions of Ena/VASP proteins and thereby inhibit their activity [69]. Importantly, the compounds inhibited cancer cell extravasation in a zebrafish model, indicating their potential for treatment of human pathologies even beyond cancer.

Indeed, transient pharmacologic inhibition of VASP may be an attractive treatment option for ischemic vascular diseases. As outlined previously, VASP-deficiency increased leukocyte infiltration, polarization, and vascular repair after ischemia [25]. Therefore, acute inactivation of VASP after myocardial infarction, stroke or occlusion of peripheral arteries constitutes a promising concept to drive therapeutic angiogenesis/arteriogenesis and therefore restore tissue perfusion. However, a leukocyte specific and/or ex vivo approach would be required to avoid systemic side effects, such as impaired endothelial barrier function or thrombotic events.

In summary, Ena/VASP proteins have been associated with various human pathologies and may therefore be interesting targets for future treatments. However, given the complex biology and the unique/redundant functions of the proteins in multiple cell types, much has to be learned to successfully translate the pre-clinical findings into new therapies.

## 17. Conclusions and Outlook

The Ena/VASP proteins are one of the most fascinating and versatile family of actin regulating proteins. In cardiovascular cells, the proteins have many important physiological functions, including the regulation of platelet activation, endothelial barrier function, cardiomyocyte contraction, conducted vasodilation and smooth muscle relaxation. Since their initial discovery more than three decades ago, much has been learned about how the Ena/VASP proteins control cell migration and cell-cell adhesions. More recently, however, evidence has accumulated for a role of this protein family in the regulation of membrane dynamics and receptor trafficking. Although the underlying molecular mechanisms are not fully understood, Ena/VASP-mediated receptor trafficking is important in the context of chemotaxis and guidance cue-mediated cell migration. The latter impacts on VEGFR2-induced endothelial cell proliferation and migration in developmental angiogenesis, CCR2-dependent leukocyte recruitment into ischemic tissue, and endothelial navigation and pathfinding downstream of ephrin/EphB signaling. It will be interesting to identify additional receptor classes and signaling pathways that are regulated by Ena/VASP-mediated membrane trafficking and to clarify, if complex formation with the receptors and/or regulation of actin dynamics is decisive for the regulation.

## Data Availability

Not applicable.
